# Effect of head group and lipid tail oxidation in the cell membrane revealed through integrated simulations and experiments

**DOI:** 10.1038/s41598-017-06412-8

**Published:** 2017-07-18

**Authors:** M. Yusupov, K. Wende, S. Kupsch, E. C. Neyts, S. Reuter, A. Bogaerts

**Affiliations:** 1 Research Group PLASMANT, Department of Chemistry, University of Antwerp, Universiteitsplein 1, B-2610 Antwerp, Belgium; 2 Leibniz Institute for Plasma Science and Technology, INP Greifswald e.V., Felix-Hausdorff-Str. 2, 17489 Greifswald, Germany; 3 Leibniz-Center for Medicine and Biosciences, Research Center Borstel, Division of Immunobiophysics, Parkallee 1-40, 23845 Borstel, Germany

## Abstract

We report on multi-level atomistic simulations for the interaction of reactive oxygen species (ROS) with the head groups of the phospholipid bilayer, and the subsequent effect of head group and lipid tail oxidation on the structural and dynamic properties of the cell membrane. Our simulations are validated by experiments using a cold atmospheric plasma as external ROS source. We found that plasma treatment leads to a slight initial rise in membrane rigidity, followed by a strong and persistent increase in fluidity, indicating a drop in lipid order. The latter is also revealed by our simulations. This study is important for cancer treatment by therapies producing (extracellular) ROS, such as plasma treatment. These ROS will interact with the cell membrane, first oxidizing the head groups, followed by the lipid tails. A drop in lipid order might allow them to penetrate into the cell interior (e.g., through pores created due to oxidation of the lipid tails) and cause intracellular oxidative damage, eventually leading to cell death. This work in general elucidates the underlying mechanisms of ROS interaction with the cell membrane at the atomic level.

## Introduction

In recent years, cold atmospheric plasmas (CAPs) are gaining increasing interest for cancer treatment, i.e., so-called “plasma oncology”^1–4^. In a recent review, Schlegel *et al*.^1^ summarize the results of several studies on plasma oncology and show the progress in the potential use of CAPs to effectively kill cancer cells (either by apoptosis or necrosis), *in vitro* as well as *in vivo*. CAP sources seem to be a powerful tool for cancer treatment, either alone or in combination with other conventional therapies. Indeed, recent experimental results showed that CAPs could enhance the effects of conventional chemotherapy even in resistant tumorous cells; the resistant cell population, if pre-treated with CAP, becomes sensitive to treatment with chemotherapy^5, 6^. Moreover, it was demonstrated that plasma treatment, both *in vitro* and *in vivo*, is able to attack a wide range of cancer cell lines without damaging their normal counterparts^1, 2^. Thus, preliminary results seem very promising. Nevertheless, the application of CAPs for cancer treatment is still in its initial stage, and there is an enormous need for a better understanding of the underlying mechanisms.

CAPs generate reactive oxygen species (ROS, e.g., OH, HO_2_, H_2_O_2_) and reactive nitrogen species (RNS, e.g., NO, NO_2_, ONOO^−^), which are generally believed to play a key role in plasma treatment^3, 4, 7^. Several studies showed that CAPs elevate intracellular ROS levels, thereby inducing oxidative damage in cancer cells, which can lead to cell death, i.e., apoptosis^3, 8^. Normal cells, on the other hand, are able to defend themselves from this harmful effect of ROS by activating multiple anti-oxidative systems that reduce the increased oxidative stress and restore the balance^9, 10^. Besides, also the RNS generated by CAP might play an important role in cancer therapy (see ref. 4 and references therein). It is suggested that the combination of nitrosative and oxidative stress induced by plasma can possibly avoid drug resistance of neoplastic cells^4^.

Although it is generally accepted that CAPs can increase the concentration of ROS and RNS (or RONS) in cancer cells, it still remains an open question whether and how these RONS from the plasma can interact with the cells and penetrate deep into the cell interior to cause oxidative stress. To answer this question, there is a need for more fundamental investigations.

Recently, Kaneko *et al*. observed the improvement of the cell membrane permeability using a cell-solution electrode CAP and they attributed this to the increase of the electric field and the density of the short-lived ROS reaching the cells in the solution^11^. Moreover, Hong *et al*. showed that plasma can deliver ROS into phospholipid (PL) membrane vesicles (i.e., synthetic cell models) without damaging the cell membrane integrity^12^. Hence, in the present work we will focus on the plasma membrane, surrounding the cell, as this is the first molecular structure of the cell that interacts with substances from outside, including plasma-generated RONS.

In first instance, we need to elucidate how the plasma species interact with the head groups of the plasma membrane. Indeed, based on free energy profiles for the transport of various ROS through a phospholipid bilayer (PLB), it can be deduced that OH, HO_2_ and H_2_O_2_ species preferably stay closer to the head groups of the PLB, whereas O_2_ molecules can accumulate at the membrane interior^13^. In ref. 14 it was reported that the barrier to O_2_ permeation into the membrane is very low, while H_2_O_2_ molecules can cross the membrane in the same manner as water (e.g., through aquaporins), as they have a hydrophilic nature. On the other hand, OH radicals are very reactive and they can immediately react with any molecule (most probably first with the head groups) when reaching the membrane^14^.

Thus, the interaction of ROS from the plasma with the head groups of the PLB forming part of the plasma membrane is the first step, which might subsequently cause (per)oxidation of the lipids. The latter can then alter the structural and dynamic properties of the membrane, which can lead to an increase in the permeability, a change in the lipid order and bilayer thickness as well as in its fluidity^15–23^.

There are already several experimental studies^15–18, 24, 25^ and a few simulation papers^20–22, 26^ devoted to a detailed investigation of the effect of lipid oxidation on the properties of the lipid membrane. Tai *et al*. studied the fluidity and structure of model membranes (i.e., giant unilaminar vesicles) under oxidative attack, using fluorescence correlation spectroscopy and Raman spectroscopy^15^. They found that OH radicals cause a significantly higher lateral fluidity of the membranes, while hydrogen peroxide has little effect^15^. An increase of the membrane disordering was observed in^16, 17^, while in^18, 24^, the opposite effect was reported, i.e., an increase of the lipid order and a drop in the membrane fluidity. The contradiction in these results can be associated with the sample preparation method and/or the depth to which the measuring probe enters the bilayer^19^, i.e., depending on the probe used, different results can be obtained^16^. On the other hand, several simulation studies, using non-reactive molecular dynamics (MD), revealed an overall increase in the membrane permeability^20^, a change in the lipid mobility in a lipid bilayer^21^ as well as pore creation and bilayer disintegration^26^ upon introduction of oxidized lipids. Similar results were also recently reported by our group^22, 23^. Moreover, we found that a higher cholesterol fraction in a bilayer with (per)oxidized lipids leads to an increase in the membrane order, and after a certain threshold (i.e., 11%) cholesterol can protect the membrane against pore formation, even when the lipid tails are fully peroxidized^22^.

Note, however, that in all these studies devoted to lipid tail oxidation, the actual oxidation process of the lipids, and more specifically of the head groups of the PLB, as well as its subsequent effect on the properties of the membrane, was not yet investigated. This forms exactly the subject of the present paper. Such a study is of great importance to find out (a) which of the RONS can react with the head groups and possibly destroy them and (b) what is the effect of the oxidized head groups on the structural and dynamic properties of the PLB. Thus, with this study, we aim to explain the onset of the lipid oxidation process. It should be mentioned here that our study is not only relevant for CAP treatment, but also to other therapies which produce ROS, such as chemotherapy, radiotherapy and photodynamic therapy.

This study requires a combination of different simulation approaches. First, we perform reactive MD simulations based on the density functional-tight binding (DFTB) method to study the interaction of ROS (namely OH, HO_2_ and H_2_O_2_) with the head groups of the PLB. It should be noted that RNS can also interact with these head groups. However, in our reactive (DFTB) MD simulations we did not observe any bond breaking and formation processes upon impact of RNS (i.e., NO, NO_2_, NO_2_^−^, ONOO^−^). This is probably due to the limited simulation time (see below), or maybe also because of the parameter set utilized in our DFTB simulations, which might not be accurate enough to describe the interactions of RNS with the head group. Subsequently, using the outcome of the reactive (DFTB) MD simulations, we apply non-reactive MD simulations based on the united-atom method to investigate the further behavior of the oxidized PLB. Finally, we also perform experiments in order to validate our simulation results.

## Description of the model systems

The plasma membrane consists of a PLB (i.e., PLs and cholesterol) with embedded proteins; they are held together mainly by non-covalent interactions^27^, and they both contribute for about 50% to the mass of the membrane. In the present work, we consider only the PLB (without cholesterol and without proteins) as a simple model system for the eukaryotic plasma membrane, since the PLB determines the bilayer thickness. Specifically, we use a PLB consisting of phosphatidylcholine molecules that are one of the four main phospholipids found in mammalian plasma membranes^27^.

**Figure 1:**
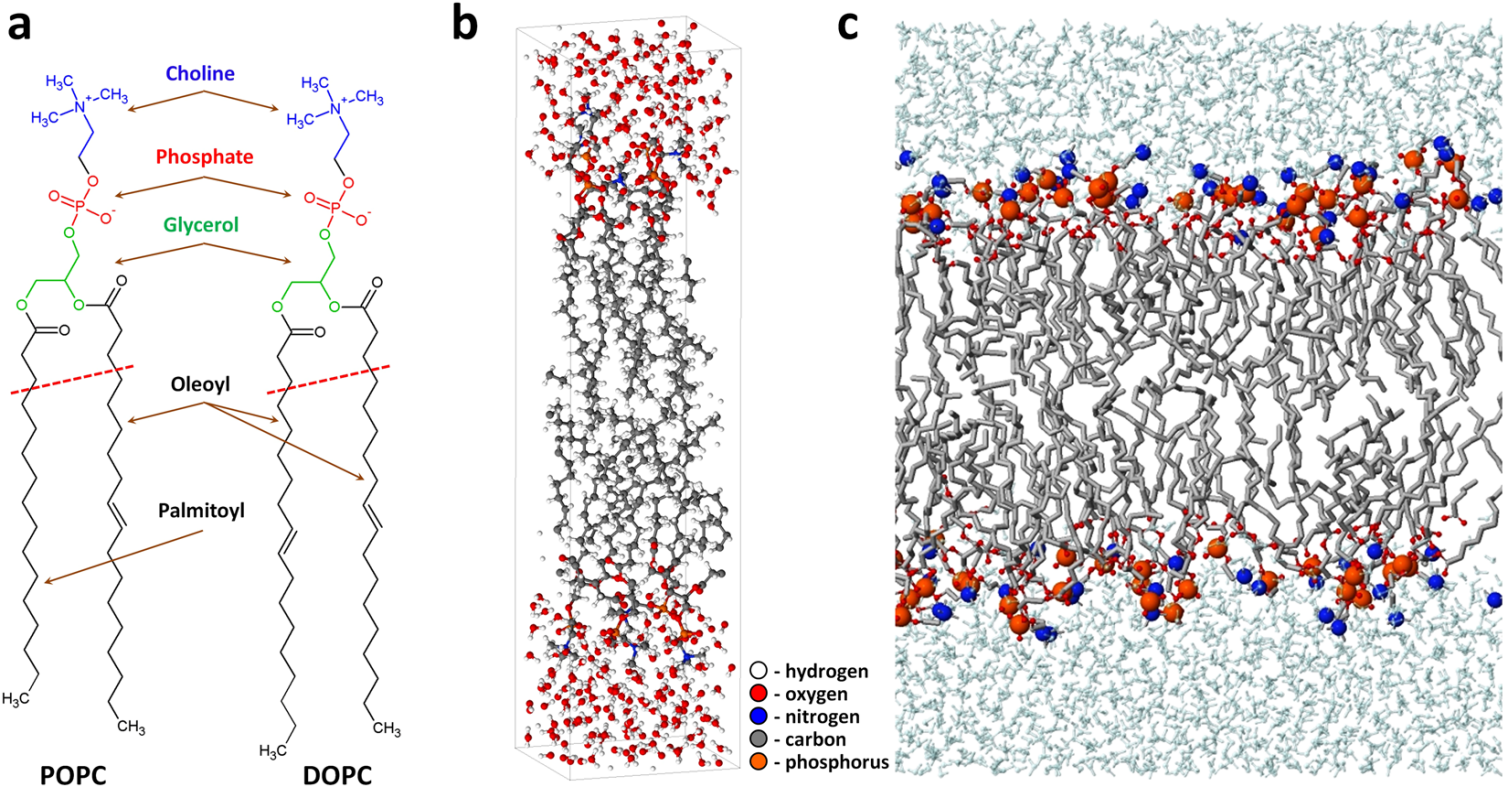
Schematic picture of the POPC and DOPC molecules (**a**) and the PLB to be treated with reactive (DFTB) MD (**b**) and non-reactive MD (**c**). The PLB in (**b**) consists of 8 DOPC molecules, i.e., 4 DOPCs (with water layer) at the top and 4 at the bottom, while the PLB in (**c**) consists of 128 DOPC molecules, i.e., 64 DOPCs (with water layer) at the top and 64 at the bottom. The structure above the red dashed lines in (**a**) is used for studying the reaction mechanisms. The water layers and lipid tails in (**c**) are shown in cyan and gray colors, respectively, and the P and N atoms are depicted with bigger beads, for the sake of clarity.

Thus, in our simulations we use two different model systems, i.e., two different PLBs, composed of 1-palmitoyl-2-oleoyl-*sn*-glycero-3-phosphocholine (POPC) and 1,2-dioleoyl-*sn*-glycero-3-phosphocholine (DOPC) molecules, respectively. Figure 1a illustrates one POPC and one DOPC molecule. They have the same hydrophilic head group (i.e., the choline, phosphate and glycerol groups) attached to two hydrophobic fatty acid tails (i.e., palmitoyl and oleoyl in the case of POPC, and dioleoyl in the case of DOPC). The oleoyl tail contains one double (unsaturated) C = C bond, whereas the palmitoyl tail remains fully saturated. Thus, DOPC and POPC only differ in the lipid tails.

The model systems presented in Fig. 1b and c are used in our reactive (DFTB) and non-reactive MD simulations, respectively. The model system of Fig. 1b is composed of 8 DOPC molecules with water layers on top and at the bottom, while the model system of Fig. 1c contains 128 DOPC molecules covered with 5120 water molecules on top and bottom layers (see below in the sections for reactive and non-reactive MD simulations, for more details).

In our experimental setup, we employ unsaturated DOPC and saturated DMPC (i.e., 1,2-dimyristoyl-*sn*-glycero-3-phosphocholine) liposomes as model systems for the biological membranes. External input of ROS including OH is imposed by CAP treatment. The membrane fluidity and the changes therein by plasma treatment are assessed by generalized polarization (GP) measurements using the fluorescent probe 6-Dodecanoyl-2-dimethylaminonaphthalene (Laurdan) for different treatment times. The dynamics of the fluidity development of the liposomes is studied by time series after plasma treatment.

In the Results and Discussion section we will show the simulation results only for DOPC and not for POPC, to allow the comparison with the experiments. Very similar results were obtained for POPC, and these are given in the Supplementary Information.

## Simulation details

### Reactive (DFTB) MD simulations

As mentioned above, our reactive MD simulations are based on the DFTB method^28^. Specifically, we use the so-called DFTB3 method^29^. Detailed information about this method as well as a parameter set used in this study is given in the Supplementary Information. As shown in Fig. 1b, in our DFTB simulations we use a model system consisting of 8 DOPC (or POPC) molecules with water layers on top and at the bottom, which is placed in a box with dimensions of ~16 Å × 72 Å × 18 Å (see the Supplementary Information for details about preparation of this model system).

It is clear that the impinging plasma species (i.e. OH, HO_2_ and H_2_O_2_) first interact with the water layer before reaching the head group of the PLB. To study their behavior in the water layer, or in other words, to determine whether these species will react with water and possibly form new species, we perform some test runs by creating a single plasma species in a small box of water (see the Supplementary Information for more details).

The total number of atoms in the system of Fig. 1b is about 2000. Because of the high computational cost of the DFTB method, we have chosen to focus on the interaction with the head groups only. Thus, instead of following the full trajectory of the ROS traveling through the water layer (which requires several tens of ps), we here consider a structure composed of a single PL molecule located in vacuum (i.e., without water layer covering the head group) and with shortened tails, i.e., with only two hydrocarbon groups after the ester groups, as illustrated in Fig. 1a (i.e., the POPC and DOPC structures shown above the red dashed line). This structure is sufficient to elucidate which reactions can possibly occur in the head group of the PLB, and which reactions have a higher probability, and are thus interesting to study in more detail (see below). Only disadvantage of using this small structure is that we cannot consider the stabilizing effect of water covering the head group (see the Results and Discussion section below). Details of the preparation of this model system are given in the Supplementary Information.

Thus, to obtain statistically valid results for bond-breaking (or bond-formation) processes and to study all possible damaging mechanisms of the PL, we perform 100 runs for each impinging species, i.e., for OH, HO_2_ and H_2_O_2_.

After the calculations with the small structure (i.e., with the single PL molecule) are finished, we use the “real” water-stabilized PLB structure presented in Fig. 1b for the analysis of the possible reactions that are obtained from the small structure.

### Non-reactive MD simulations

The structures obtained from the reactive (DFTB) MD simulations are employed in the non-reactive MD simulations to study their further behavior, i.e., the change in structural and dynamic properties of the PLB upon oxidation of the head groups. For this purpose we apply so-called united-atom MD simulations. This method allows to investigate larger systems (up to 100,000 atoms) and longer time-scales (hundreds of ns)^30, 31^, which are clearly not accessible in an all atom-model like DFTB.

In the united-atom approach, each hydrocarbyl group (i.e., CH_3_, CH_2_ and CH) is treated as a single group, without internal structure. Thus, the united-atom representation is adopted for the apolar alkyl chains as well as for the hydrocarbyl groups in choline and glycerol, which reduces for instance the size of the DOPC molecule, containing 138 atoms, to a system of 54 particles. The polar H-atoms (in the water molecules or in an alcohol-group) remain, however, as separate atoms.

In total, 9 different model systems are prepared for DOPC, and 5 for POPC, including intact and oxidized PLBs, with two different oxidation products at various concentrations (see below), all containing 128 lipids as well as 5120 water molecules surrounding them in the top and bottom layers. Our reactive (DFTB) MD simulations showed that two oxidation products (called here OX1 and OX2) are most abundantly formed (see below in the Results and Discussion section, for more details). On the other hand, the experimental results revealed only the OX2 product, together with products of lipid tail oxidation, which we call ALD, as more prominent oxidation products (see below). Thus, in our non-reactive MD simulations, we describe the effects of these two oxidation products (i.e., OX2 and ALD) at five concentrations, ranging from 0% (intact PLB) to 100% (fully oxidized PLB). More details about the concentrations of the oxidation products as well as the preparation of these oxidized PLB systems are given in the Supplementary Information.

The simulations are carried out using GROMACS 4.6 software, applying the classical united-atom GROMOS 43A1-S3 force field^32^, which contains parameters for a wide variety of lipids, including DOPC and POPC. To validate the force field for this application, we compared our calculated values of the surface area per lipid and the bilayer thickness, which define typical properties of the bilayer, for the intact PLB, with results obtained from experiments and computational studies. Our computational results are well within the range of data reported in literature (see Supplementary Table S1).

It should be mentioned that for the oxidized PLs, new force field parameters are required for the newly formed bonds and associated angles and dihedrals. Most of these parameters are not available in the applied force field, or in other force fields from literature. Therefore, these parameters had to be determined first, by means of density functional theory (DFT), as will be explained in the next section. The parameters of the ALD oxidation products are obtained from ref. 20.

### DFT simulations

We employ DFT calculations to obtain new parameters for the non-reactive force field by carrying out a fitting of some standard potential energy functions to the DFT energy. Note that for the newly formed bonds, angles and dihedrals, we try to use as many parameters as possible that already exist in the parameter set of the original force field (i.e., GROMOS 43A1-S3), i.e., we perform the parametrization (or fitting) procedure only for those bonds or angles or dihedrals which are not included in the force field. In this way, we keep the parameter set as native as possible. Thus, we implement 16 new parameters to the force field. All new parameters are given in Table S2 of the Supplementary Information, and more explanation about the fitting procedure can be found there.

## Experimental setup

### Plasma Source

For the experiments, liposomes are treated with plasma-activated liquid. The plasma source used is a so-called kINPenSci^33^. The plasma jet is a needle type discharge in a dielectric tubing of 1.6 mm inner diameter. The grounded ring-shaped electrode is located at 2 mm distance from the dielectric nozzle exit. The plasma source is operated with argon as feed gas (purity 5.0) with a flux of 3 standard liters per minute (slm). An approximately 10 mm long visible plasma plume is driven out of the jet nozzle. The kINPenSci is based on the design of the commercially available kINPen, except that additional possibilities are available to measure electric signals of the excitation frequency. The kINPen is a plasma jet that is also used – in the form of the kINPen MED^34^ – in wound healing^35, 36^ and for cancer studies *in-vitro* (see e.g. ref. 37) and *in-vivo* ^38^. The kINPen is a source of ROS^39–41^ and in certain cases RNS^42^. It generates OH in the gas phase^39^ and in the liquid phase^43^.

To provide stable and reproducible plasma treatments, the plasma source including gas flow is switched on at least 1 h before treatment. By this procedure, the influence of variations of feed gas humidity can be minimized, which has a big impact on the produced plasma species and on the behavior of the plasma-treated cells^44^. Furthermore, a so-called gas-shield is used to prevent influences of changing natural ambient air on the effluent – gas interaction^45^. The used shielding gas mixture is 1.25 slm O_2_ and 3.75 slm N_2_, so a mixture of 25% O_2_ and 75% N_2_. The shielding gas is switched on at least 1 h prior to treatment.

### Preparation of Liposomes

A stock solution of 2.5 mM lipids in chloroform is prepared from commercially available 10 mg/ml chloroformic lipid solutions (Avanti Polar Lipids, Alabaster, Alabama, USA). A solvent free lipid film is prepared by drying 400 µl stock solution in a glass vial under gentle nitrogen stream for 2 h. A 250 µM liposome suspension is prepared from the film by addition of 4 ml aqueous buffer, containing 100 mM KCl, 5 mM magnesium chloride hexahydrate and 5 mM HEPES at pH 6.7. Lipid detachment and liposome formation is achieved by ultrasonification at 70 °C, followed by 30 min incubation at 4 °C. The thermic cycle is repeated twice.

### Plasma treatment procedure

Plasma treatment of liposomes is accomplished by placing 5 mL of liposomal solution in a 60 mm diameter plastic petri dish under the plasma jet’s nozzle with a distance of 9 mm to the aqueous surface. (Trasdingen, Switzerland). Positioning and moving of the jet is performed automatically by an XYZ-positioning stage (Hylewicz CNC-Technik, Geldern, Germany) above the liquid surface on a predefined meandered path to ensure a thorough and homogeneous treatment of the sample. The petri dish is immediately closed after treatment. For the mass spectrometry of both unsaturated DOPC and saturated DMPC lipids, 250 µl liposome solution is mixed with 250 µl high purity water and treated in 1 cm^3^ glass vials with the same device for 3 min.

### Laurdan Assay

Membrane phase states (gel- or liquid-phase) can be detected by measuring the penetration of water molecules into the bilayer, which strongly correlates with the packing of the phospholipids. This can be done by using 6-Dodecanoyl-2-dimethylaminonaphthalene (Laurdan), a lipophilic fluorescent probe that readily inserts into membranes. Laurdan is located at the *sn*-1 acyl chain^21^. The hydrophobic tail allows for integration parallel to the fatty acids of the lipids due to strong Van-der-Waals and hydrophobic interactions^46^. In this way, the hydrophilic and fluorescent naphthalene component aligns with the phospholipid glycerol backbone and points towards the aqueous surrounding. The increase in the dipole moment caused by the excitation of the Laurdan molecule triggers a reorientation of the water dipoles in the immediate vicinity of the probe. This interaction requires energy, resulting in a dipolar relaxation, meaning that Laurdan loses energy in its excited state. As a consequence, the maximum of the emission spectrum is shifted towards higher wavelengths from 422 nm to 490 nm if the excitation is 350 nm. This Stokes-shift correlates to head group hydration and mobility^21^. The detection of membrane phase transition is considered constant, as the fluorescent properties of Laurdan are independent of phospholipid head groups or lipid linkage^47^. The ratio of emission intensities at 422 nm and 490 nm can be used to calculate the so-called generalized polarization (GP) value, a direct measure of water penetration.

Laurdan was purchased from Life Technologies (Darmstadt, Germany) and was dissolved in 99.5% ethanol. In the present work, Laurdan is added to the respective 20 µM liposomal solution to yield a lipid to Laurdan ratio of 300:1, respectively. The working solution is vortexed and incubated for 30 min in the dark at room temperature. 5 mL of the sample are placed in a petri dish and treated with the kINPen plasma source for 5, 10, or 30 min as described above. The samples are subsequently incubated for 10 min at room temperature.

2 mL of the sample are analyzed using a high precision cell (special optical glass, light path 10 mm; Hellma Analytics, Müllheim, Germany), which is placed in a temperature controlled sample chamber of a spectrofluorometer (Fluorolog-3, Horiba Jobin Yvon Inc., Edison, New Jersey, USA). The excitation wavelength is 350 nm and the emission maxima are measured at 422 nm and 490 nm at constant 21 °C. Two untreated samples are measured. The treated sample is measured every three minutes over a course of three hours. For the determination of membrane fluidity changes, the temperature is gradually increased from 20 °C to 40 °C in steps of 2 °C. Laurdan emission is recorded 10 min after temperature change, in order for the sample temperature to equilibrate.

### High resolution mass spectrometry

After treatment, liposomes are collected from the aqueous phase by CHCl_3_ extraction. The resulting lipid solution is diluted six-fold for direct infusion into a TripleTOF5600 high resolution mass spectrometer (MS) by water-methanol-formic acid (positive mode) or water-methanol-ammonia (negative mode). Using electrospray ionization (ESI) at appropriate conditions (see below), a full scan between m/z 50 and 1000 is recorded. Base peak and monoisotopic peaks are manually annotated and screened for potential covalent modifications related to the plasma treatment.

The direct infusion rate is 10 µl s^−1^, the sample is dissolved in 80% methanol MS grade, 10% CHCl_3_, 10% water, 0.1% formic acid. The capillary temperature is 200 °C and the ionization potential is +4000 V. The depolarization potential is 10 V, and the collision energy is also 10 V.

## Results and Discussion

### Reactive (DFTB) MD results

#### ROS behavior in the surrounding water layer

As mentioned in the section on Reactive (DFTB) MD simulations above, the impinging ROS (i.e. OH, HO_2_ and H_2_O_2_) first interact with the water layer surrounding the PLB, before interacting with the head group of the PLB. Therefore, it is necessary to know which of the ROS can penetrate through the water layer and effectively reach the head group. We observed no bond breaking or formation events in the case of HO_2_ and H_2_O_2_, so these species can freely move through the water layer. The OH radicals, on the other hand, can react with water, exchanging a hydrogen atom and forming again the same species (i.e., a new OH radical and a water molecule), a process which is continuously repeated during the transport of OH radicals through the water layer. The behavior of the ROS in a liquid layer was also studied in detail in our previous work by means of classical reactive MD simulations applying the ReaxFF potential^48^, and we found that OH, HO_2_ and H_2_O_2_ can travel deep into the water layer and eventually reach the surface of the biomolecule. The same behavior is thus predicted here with the DFTB method. The only difference is that in our previous work the HO_2_ radicals reacted with a H_2_O molecule, forming a superoxide ion (O_2_^−^) and a hydrated proton (H_3_O^+^), which recombine immediately back (within a few fs) to create the reactants again (see ref. 48 for more details), while this process was not observed in our current DFTB simulations. It was reported based on a DFT/MM approach that HO_x_ radicals are very good proton donors and weak proton acceptors^49^, and the hydrogen bonds between HO_x_—H_2_O are much stronger in solution than in gas phase, pointing towards the stability of HO_x_ species in water^49^. In this sense, the DFTB-MD results on HO_2_ seem to be more accurate than the ReaxFF-MD results. However, this is not so important, as the backward reaction found in the ReaxFF-MD results is so fast, and thus, both methods predict that the HO_2_ radicals can penetrate through the water layer. Moreover, Moin *et al*. have revealed using *ab initio* MD simulations that H_2_O_2_ molecules are also very stable in water due to the (at least four) hydrogen bonds found between H_2_O_2_ and water molecules^50^. Recently, Verlack *et al*. investigated the behaviour of OH radicals and O atoms in bulk water applying reactive (DFTB) MD simulations^51^. They concluded that the transport of OH radicals in water is not only governed by diffusion, but also through an equilibrium reaction of H-abstraction with water molecules, in line with our previous ReaxFF-MD study. They also studied the self-interaction of two OH radicals and observed some distinct reactions^51^. We can thus conclude that all of the ROS, i.e., OH, HO_2_ and H_2_O_2_, are in principle able to reach the PLB, after travelling through a water layer.

#### ROS interaction with the head groups of the PLB

As mentioned in the Introduction, we focus on the interaction of the ROS with the head groups of the PLB, and not with the lipid tails, as much less is known in literature about the products formed upon interaction of ROS with the head groups. To find out which reactions occur, we first performed reactive (DFTB) MD simulations using a single PL molecule with the shortened lipid tails (see above in the section on Reactive (DFTB) MD simulations). Like in the water case, HO_2_ and H_2_O_2_ molecules do not react with the head group. They only have non-bonded interactions with the structure. To check this in the “real” structure (i.e., the PLB consisting of 8 PLs, see Fig. 1b) with water layers on top and bottom, we created two of the same plasma species (e.g., two HO_2_ radicals) in the water layers, i.e., one on top and another in the bottom, and we simulated their behavior for 5 ps (i.e., 10^4^ iterations). Due to the severe computational cost of DFTB, we positioned the plasma species closer to the head groups. Again no bond breaking of the head groups upon interaction with these species (i.e., HO_2_ and H_2_O_2_) was observed.

On the other hand, the OH radicals do react with the head groups of the PLB, leading to the cleavage (or formation) of some bonds. These reaction mechanisms will be discussed below in more detail.

#### Reaction mechanisms upon OH interaction with the head groups of the PLB

As mentioned in the section on Reactive (DFTB) MD simulations, we performed 100 MD runs using a small structure, to gain some limited statistics, as well as to study the most probable reaction mechanisms of OH interacting with the head groups of the PLB. We observed six reaction mechanisms, called OX1, OX2, …, OX6 (see the Supplementary Information) and found that all of them are initiated by H-abstraction from (different parts of) the head groups, but few of them give rise to further bond breaking. We subsequently investigated all of these reaction mechanisms in the “real” water-stabilized PLB structure and observed the same reaction mechanisms, but the breaking of important bonds (if any, see below) was much slower, especially in the case of OX1, which is due to the stabilizing effect of water covering the head groups. Therefore, we need to consider these reaction mechanisms with caution and validate them with experimental results, which is the case in this study (see below in the Experimental validation section).

**Figure 2:**
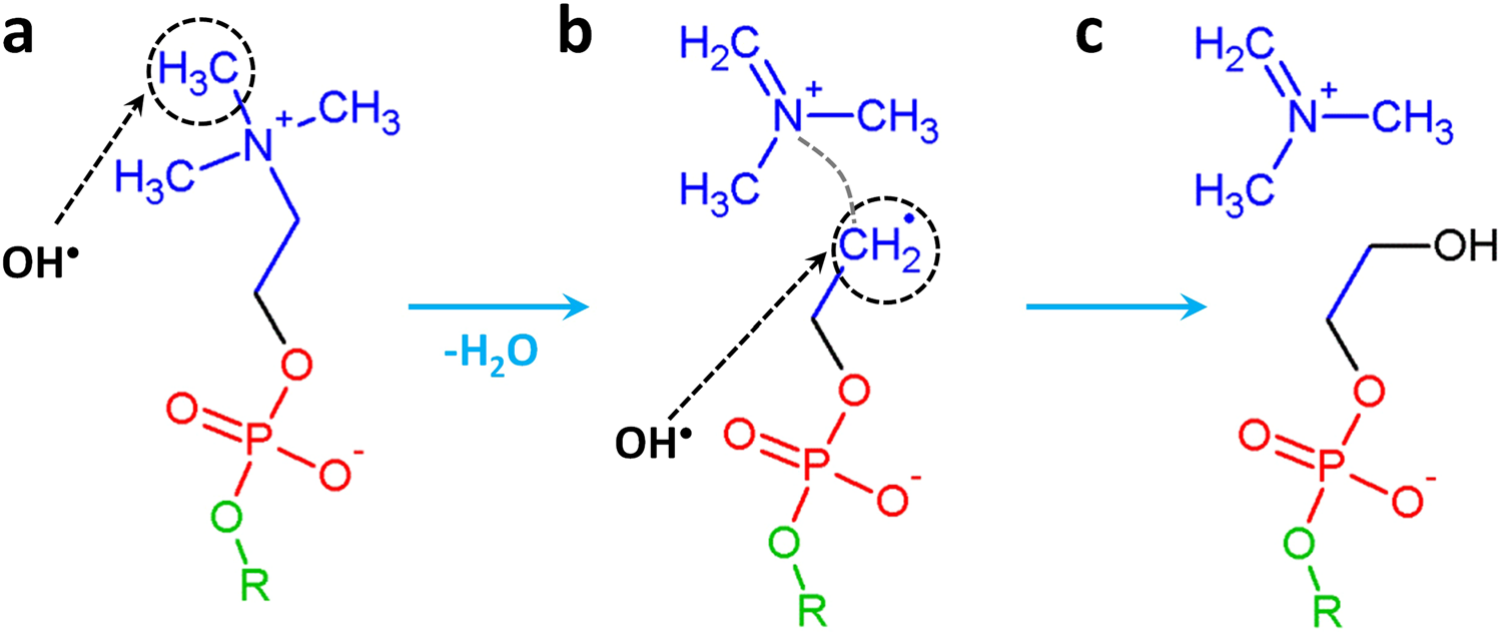
Breaking mechanism of the C-N bond upon impact of an OH radical. This impact first results in H atom abstraction (see black dashed circle in (**a**)) and the formation of a water molecule, followed by the cleavage of the C-N bond (see gray dashed line in (**b**)), and thus the detachment of the NC_3_H_8_^+^ group as well as the formation of a radical site (see black dashed circle in (**b**)). A new OH radical can then react with this site, resulting in (**c**) the formation of an alcohol group.

We focus here only on two mechanisms which lead to the detachment of some parts of the PL head groups, and are thus important for the investigation of the longer-term behavior of the PLB (see the Non-reactive MD results section). Figure 2 illustrates one of these interaction mechanisms. Note that this reaction mechanism was observed most in our simulations (i.e., in 47% of the cases, although we need to consider this percentage with caution, due to the limited statistics).

The OH radical first abstracts a H atom from the methyl group of choline (see dashed circle in Fig. 2a) forming a water molecule. This leads to the breaking of a C-N bond (see gray dashed line in Fig. 2b) and the formation of a double C=N bond, resulting in the detachment of the NC_3_H_8_^+^ group, as well as the creation of a dangling bond at the C atom (see dashed circle in Fig. 2b).

Subsequently, a new OH radical can either react with this C radical (see Fig. 2b), forming an alcohol group (see Fig. 2c) or abstract the H atom from the CH_2_ group positioned beneath this radical, forming a stable double C=C bond. To check which of these two reactions is energetically more favorable, we performed DFT calculations. The calculated reaction energies revealed that the formation of the alcohol group (as depicted in Fig. 2c) is energetically slightly more favorable (see Fig. S2).

**Figure 3:**
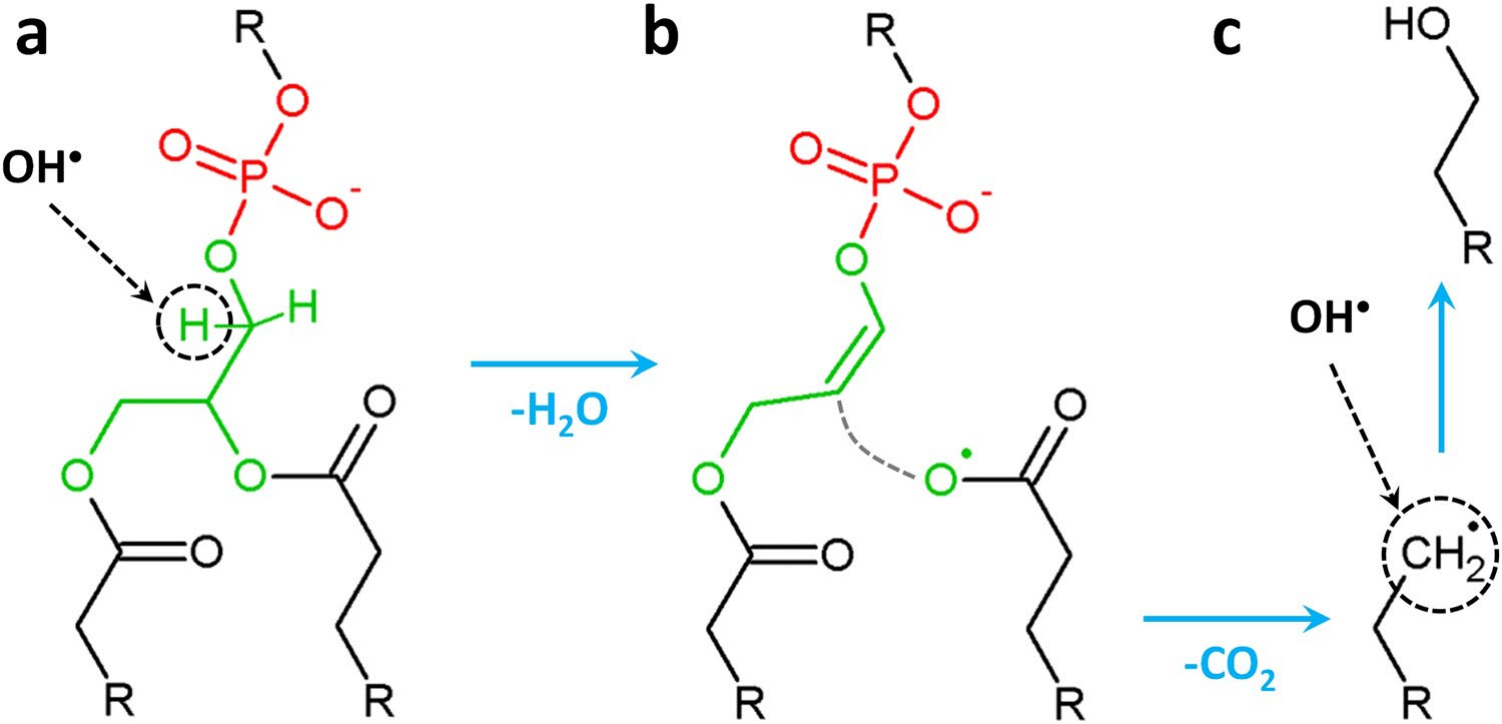
Breaking mechanism of a C-O bond upon impact of an OH radical. The OH radical abstracts a H atom (see black dashed circle in (**a**)) leading to the formation of a water molecule. This subsequently results in the formation of a double C=C bond, the cleavage of a C-O bond (see gray dashed line in (**b**)) and the detachment of a CO_2_ molecule, leaving behind a radical site (see black dashed circle in (**c**)). A new OH radical can then react with this site, forming an alcohol group (**c**).

The second interaction mechanism, which is observed in 6% of the cases, leads to the detachment of one of the tails of the PLB, and is therefore also highly important for the longer-term simulations. This mechanism is schematically represented in Fig. 3.

After the H-abstraction from CH_2_ of glycerol (see dashed circle in Fig. 3a), a water molecule and a double C=C bond are formed, followed by the detachment of the whole oleoyl chain (see Fig. 3b and cf. also Fig. 1a). Subsequently, a CO_2_ molecule detaches from this oleoyl chain, leaving behind a radical site (see dashed circle in Fig. 3c). A new OH radical can then react with this radical site, forming an alcohol group.

To summarize, we distinguish two reaction mechanisms of OH radicals interacting with the head groups, which lead to the destruction of the PL molecules and are therefore important for studying the subsequent longer-term effects of these oxidized PLs on the properties of the PLB. We did not consider the other reaction mechanisms (i.e., OX3–OX6, see the Supplementary Information), since they either do not lead to structural damage of the PLs (and are therefore unimportant in the context of the longer-term simulations) or their consequences are very similar to the mechanisms shown above and therefore we expect that their long-term consequences are also similar. A more detailed discussion about the other reaction mechanisms can be found in the Supplementary Information (see Fig. S3). As mentioned above, we need to take aforementioned reaction mechanisms (i.e., OX1 and OX2) into account with caution, as we did not consider the stabilizing effect of water surrounding the head groups. Therefore, we perform some experimental validation (see next section) in order to decide whether these oxidation products should be used in our further non-reactive MD simulations to study the longer-term behavior of the oxidized PLB.

### Experimental validation: Results of the high resolution mass spectrometry

**Figure 4:**
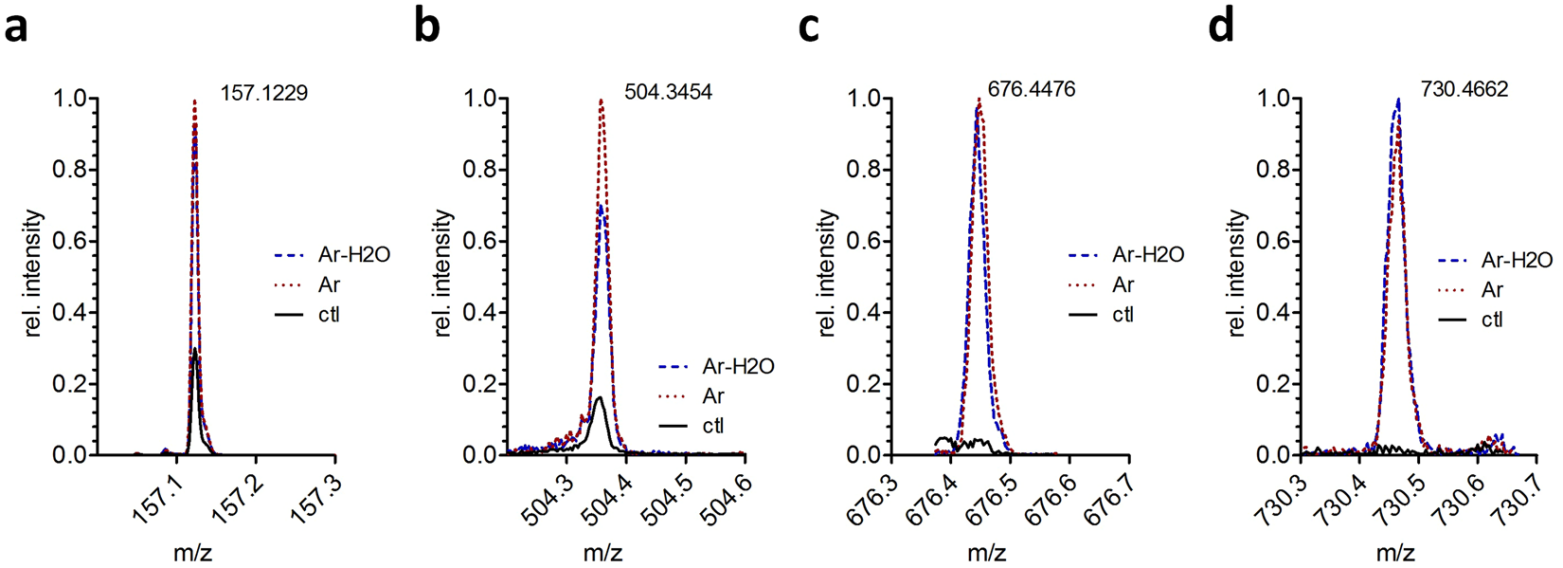
High resolution mass spectrometry peak intensities of (**a**) nonanoic acid (157.1229 m/z, negative mode, DOPC, product of ALD mechanism), (**b**) OX2 product of DOPC (504.3454 m/z, positive mode), (**c**) ALD product of DOPC (676.4476 m/z, positive mode) and (**d**) dimer resulting from OX2 product of DOPC (730.4662 m/z, positive mode), all normalized to the respective most intense signal.

Using mass spectrometry as an orthogonal technique, we searched for covalent changes to the molecular structure of both unsaturated DOPC and saturated DMPC after the plasma treatment. In positive mode, the molecular ion of DOPC was detected at 786.6015 m/z (0.3 ppm error, base peak, see Fig. S5). The molecular ion of DMPC was detected at 678.5079 (0.5 ppm error, base peak, see Fig. S6). Prominent ions beside the base peaks and their isotopes were complexes with alkali and earth alkali ions. No principal changes in the mass spectra of the respective lipids were observed, indicating that the impact of the reactive species was limited, e.g. by the ratio [lipid molecules]/[reactive species] and protective effects of the liposomal structures, especially regarding side chain oxidation (i.e., ALD oxidation, see below). Yet, a number of smaller signals indicative for plasma treatment were found for both DOPC and DMPC (Fig. 4).

**Figure 5:**
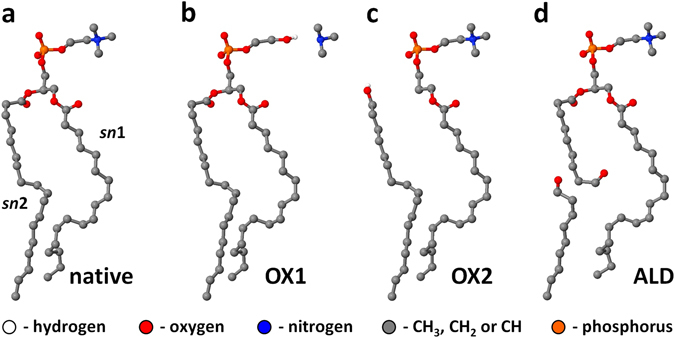
Schematic representations of the native DOPC molecule (**a**), together with its oxidation products ((**b**), (**c**) and (**d**)). The reactive (DFTB) MD simulations reveal OX1 (**b**) and OX2 (**c**) as mostly observed oxidation products of head group oxidation, whereas the experiments suggest OX2 (**c**) and ALD (**d**) to be more prominent oxidation products. All the hydrocarbyl groups (i.e., CH_3_, CH_2_ and CH) are now represented by united-atoms. The same oxidation products as observed in the reactive (DFTB) MD simulations are also found for POPC.

Two routes of lipid modifications were experimentally observed: oxidation of the lipid side chain (which we call ALD, see Fig. 5d below) and elimination of *sn2* tail (corresponding to OX2, see Fig. 5c below). Side chain oxidation was only observed when using unsaturated DOPC lipids and manifested in the presence of two products, a short fatty acid (nonanoic acid, 157.1229 m/z, negative mode, see Fig. 4a) and a residual phosphatidylcholine bearing an aldehyde function *sn1* or *sn2* (9-oxo nonanoic acid at *sn1/2*, 676.4476 m/z, positive mode, see Fig. 4c). According to the proposed mechanism from MD simulations^20, 22, 26^, the initial product of ALD is the corresponding nonanal (see short chain in Fig. 5d), which cannot be detected by ESI mass spectrometry. Via secondary oxidation, the detectable nonanoic acid arises. For saturated DMPC, missing an attackable allyl position, similar products were not detected. Beside the side chain, covalent changes in lipid structure can occur at the polar head group, because the high electron density and the presence of heteroatoms allow numerous chemical reactions. In our MD simulations, two dominant reactions were initially predicted: OX1 and OX2. OX1 reflects the attack of hydroxyl radicals at the choline residue, leading to the loss of the amino group. However, the corresponding product, phosphatidylglycol (742.5149 m/z; 634.4210 m /z, negative mode), was neither detected for DOPC nor DMPC. Either OX1 is not occurring after plasma treatment, or the products withdraw from detection for their physicochemical properties (lower polarity, bad ionization efficacy), or concentration. In contrast, the OX2 mechanism reflects an elimination of a fatty acid chain at the glycerol moiety, leaving a propendiol group. The respective products (propendiol-phosphatidylcholines) were detected in positive mode for DOPC (504.3454 m/z, see Fig. 4b) and DMPC (450.2984 m/z). Additionally, a dimeric structure related to the OX2 propendiol substructure was found for DOPC (C_34_H_72_N_2_O_10_P_2_ 730.4662 m/z, positive mode, see Fig. 4d) and DMPC (C_30_H_66_N_2_O_10_P_2_ 676.4116 m/z; positive mode). Due to limitations of fragmentation modes and signal intensity, the structure could not be completely established but the molecular composition was determined with a sub-ppm error. Presumably, two propendiols stabilize by eliminating the double bonds and expelling a further fatty acid side chain, creating a molecule bearing two phosphatidylcholine head groups, one fatty acid, and a glycerol – propandiol ether structure. This product was found predominantly in DOPC, and the fatty acids (oleic acid, myristic acid) were found for DOPC and DMPC (negative mode). Signals for both OX2 and ALD oxidations were observed with intensities almost independent from the water admixture to the working gas, although this enhances the flux of H_2_O_2_ and OH radicals. However, a slight superiority for Ar-H_2_O in OX2 was observed (Fig. 4d).

In summary, as is clear from the ESI MS results, it seems that OX2 and the formation of aldehyde groups (which we call here ALD, see next section) are more prominent. Note that although the experiments and simulations cannot be directly compared due to the vastly different time scales, they certainly provide a complementary view, and the experiments seem to validate our calculation results on OX2 formation.

### Non-reactive MD results

#### Influence of the concentration of the oxidized lipids

As is clear from the previous sections, the reactive (DFTB) MD results reveal two important oxidation products of the head groups (i.e., OX1 and OX2), whereas the MS results suggest OX2 and ALD (i.e., the formation of aldehyde groups in one of the lipid tails) as more prominent oxidation products. A schematic picture of these oxidation products is given in Fig. 5.

Thus, in our non-reactive MD simulations we consider only OX2 and ALD oxidation products and investigate their long-term effects on the properties of the PLBs. For this purpose we analyze some important properties of the bilayers:

1. *The surface area per lipid*, determined by dividing the surface area of the bilayer (averaged over the 100 ns, by sampling the data after every 20 ps) by the number of lipids of one leaflet of the PLB (i.e., L_x_ × L_y_/64, where L_x_ and L_y_ are the *x* and *y* dimensions of the PLB, respectively, and 64 is the number of lipids present in one layer).
2. *The bilayer thickness*, calculated by averaging all distances (*z*-components) between the phosphate groups of the two opposite layers of the PLB. This distance is also averaged over the 100 ns simulation time, by sampling the data after every 10 ps.
3. *The deuterium order parameter, S*_*CD*_, which is a measure for the order of the lipid tails in the bilayer. The degree of ordering of the lipid tails can also be influenced when the head groups are oxidized (see below). More detailed information about *S*_*CD*_ can be found in ref. 22.

**Figure 6:**
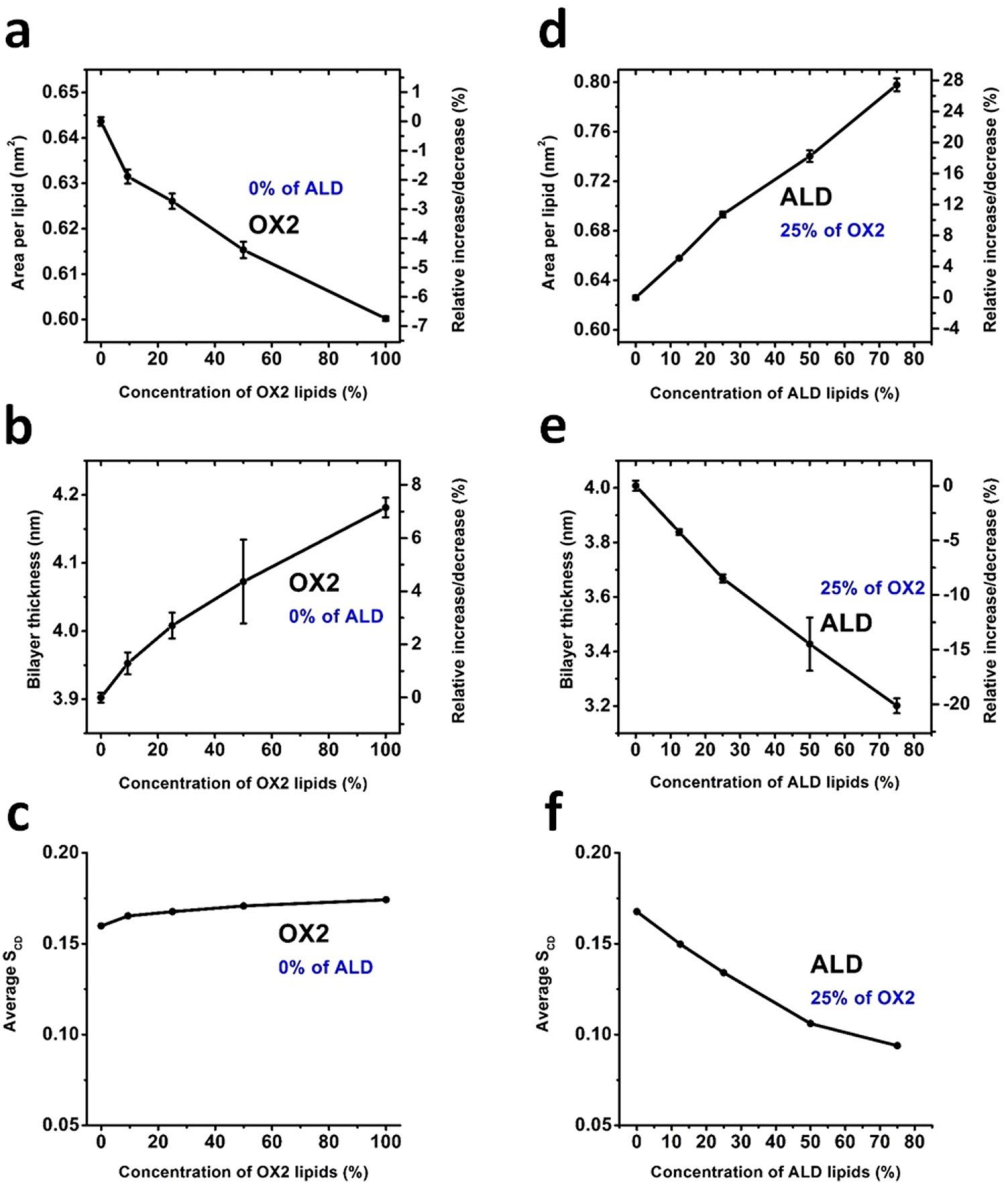
Surface area per lipid (**a**) and (**d**), thickness of the bilayer (**b**) and (**e**) and average deuterium order parameter (**c**) and (**f**), as a function of the concentration of the oxidized PLs, for two types of oxidation products, for the DOPC PLB. Note that (**a**)–(**c**) illustrate the properties of the DOPC PLB with only OX2 oxidation, whereas (**d**–**f**) show the results of combined effects of ALD and OX2. In this case the concentration of ALD ranges between 0–75%, while keeping that of OX2 at 25%.

The surface area per lipid, the bilayer thickness and the deuterium order parameter are plotted in Fig. 6 as a function of the concentration of the oxidized PLs. These graphs are presented for the DOPC bilayer, but very similar results were obtained for the POPC bilayer (see Fig. S4a,b).

Based on the experimental results given in the next section, we consider two cases. First, we analyze the above mentioned properties of the PLB for OX2 oxidation, changing its concentration from 0% (native PLB) to 100% (fully oxidized PLB). This case can be related to the direct treatment of the DOPC vesicle with the plasma source (see the discussion in the next section). Secondly, we examine the properties of the PLB applying both oxidation products (i.e., OX2 and ALD) at the same time, to study their combined effect. In this case, we keep the concentration of OX2 at 25%, which is in our opinion more realistic, whereas changing the fraction of ALD from 0 to 75%. The latter concentration (i.e., 75% ALD and 25% OX2) again corresponds to the fully oxidized PLB. This case can correspond to the post treatment of the DOPC vesicle with plasma.

It is clear from Fig. 6a that upon increasing the concentration of OX2, the surface area per lipid decreases by maximum ~7%, whereas it increases in the case of ALD + OX2 up to ~28% (see Fig. 6d). On the other hand, the bilayer thickness increases upon OX2 oxidation (up to 7% for full oxidation, see Fig. 6b), whereas it decreases in the case of ALD + OX2, but this decrease is again more pronounced (see Fig. 6e). Finally, the calculated average *S*_*CD*_ shows that when OX2 takes place, the order of the tails somewhat increases, whereas it again considerably decreases in case of ALD + OX2 (see Fig. 6f). Thus, we obtain opposite results for OX2 and ALD + OX2 for all calculated parameters, but the effects are more pronounced for ALD + OX2.

**Figure 7:**
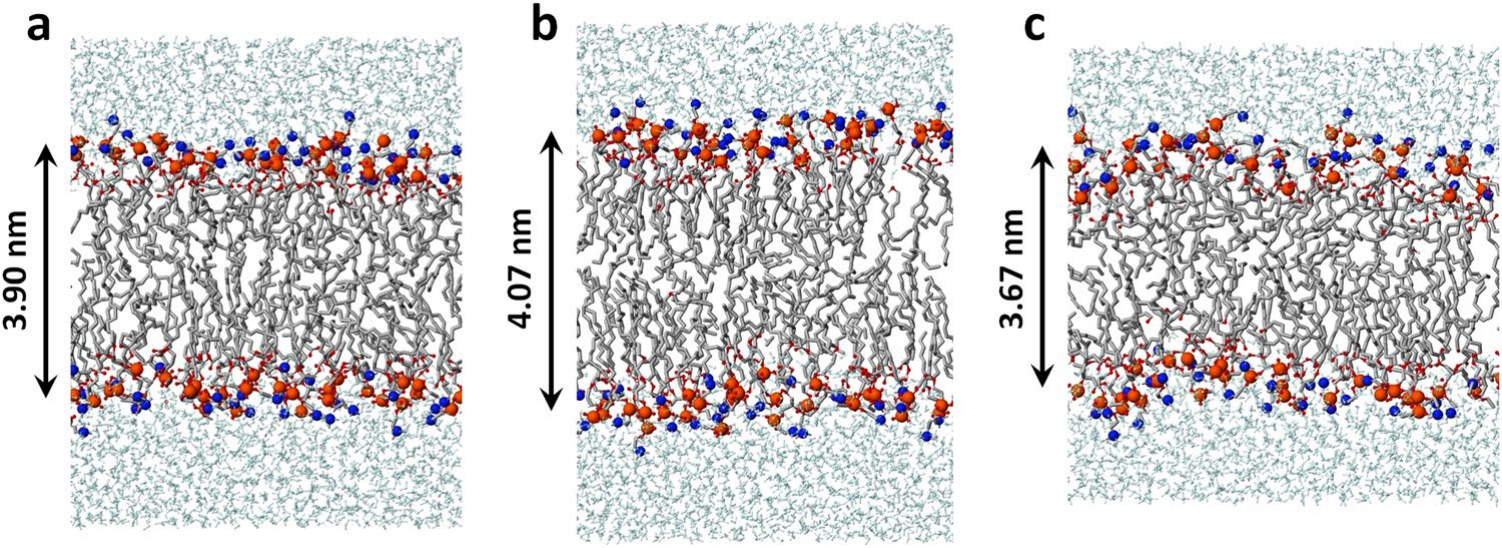
Schematic picture of the original DOPC PLB (**a**), and the DOPC PLB with only oxidized head groups (i.e., 50% OX2 (**b**)) as well as with oxidized head groups and lipid tails (i.e., 25% OX2 and 25% ALD (**c**)), cf. Figure 6 for the values of the bilayer thickness. The color legend is identical to Fig. 1.

We believe that the reason for these two opposite phenomena is the following. In the case of OX2, the detached lipid tails (see Fig. 5c) exert a stiffening effect on the lipids (see Fig. 7b), similar to the action of cholesterol (see ref. 22), although the effect is not so pronounced as in the case of cholesterol. This stiffening effect leads to a slight increase in the order of the tails (see Fig. 6c), thereby slightly increasing the bilayer thickness (but to a lower extent than the decrease in the case of ALD + OX2, cf. Fig. 6b and e). As the bilayer thickness slightly increases, the probability of (per)oxidation of the lipid tails will slightly decrease. Thus, the bilayer becomes slightly more rigid upon OX2 oxidation. In the case of ALD + OX2, the detached small aldehyde tails (see Fig. 5d) can freely move within the lipid region as well as towards the head group (see Fig. 7c). Moreover, shortened lipid tails (see Fig. 5d) can also bend towards the head group, thereby increasing the average area per lipid (see Fig. 6d). At the same time, this leads to a decrease of the bilayer thickness (see Figs. 6e and 7c) as well as a drop in the lipid order (see Fig. 6f) upon ALD oxidation. The long-term effect of the latter was recently studied in refs 22 and 23, and it was observed that this can even lead to pore formation, in accordance with previous MD results^20, 26^.

Note that similar results of OX2 oxidation as shown here for DOPC were also obtained for the POPC bilayer (see Fig. S4a,b). Moreover, we have also carried out simulations for a fully saturated lipid, more specifically for a DPPC (i.e., dipalmitoyl phosphatidylcholine) PLB. The results are given in the Supplementary Information. It is clear that similar changes in the surface area per lipid and the bilayer thickness as a function of the concentration of the OX2 oxidation are obtained, but the relative changes are more pronounced than in case of DOPC and POPC (see Fig. S4c,d).

In summary, the oxidation products OX2 and combined ALD + OX2 lead to opposite results for all calculated parameters, but the effects are more pronounced for ALD + OX2. Already 25% of ALD oxidation leads to an increase of the surface area per lipid and a decrease of the bilayer thickness by around 10% (see Fig. 6d,e), even when 25% of OX2 takes place in the PLB. Thus, our calculations predict an overall increase in area per lipid and a drop of the bilayer thickness upon equal oxidation of the head groups and lipid tails, thereby decreasing the lipid order, which eventually leads to an increase of the bilayer fluidity. The change in fluidity was also studied by experiments to validate our simulation results and similar results were observed (see next section).

It should be mentioned here that despite the fact that the effect of OX2 is less pronounced, its oxidation byproduct, namely the phospholipid structure with a single lipid tail (see oxidized structure with acyl *sn1* chain in Fig. 5c) may play an important role in cancer treatment. Indeed, the chemical structure of this byproduct is very similar to the structure of so-called alkylphospholipids (APLs, e.g., LysoPC^52^), which are proven to be effective in cancer treatment^52–54^. Unlike most anticancer drugs, APLs do not target DNA, but they insert in the plasma membrane and subsequently they induce a wide range of biological effects, eventually leading to cell death. The effectiveness of APLs is mediated by changes (i.e., destabilization) in the lipid domains and displacement of signaling proteins. Therefore, the oxidation byproduct of OX2 (generated by the CAP induced ROS) might destabilize the lipid domains in the cell membrane, which may ultimately result in cell death. For more information about APLs and their apoptotic effects, we refer to some reviews^52–54^ and references therein.

### Experimental validation: Results of the Laurdan assay

**Figure 8:**
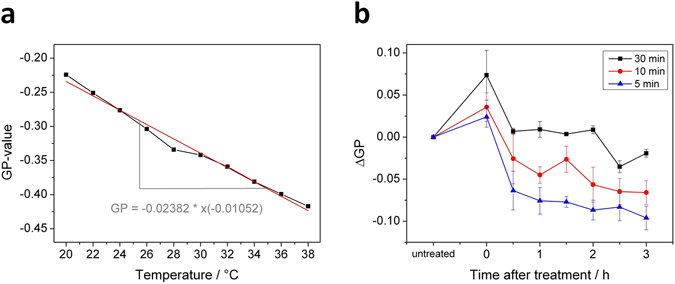
(**a**) Effect of membrane bilayer temperature on Laurdan generalized polarization (GP). DOPC liposomes were labeled with Laurdan to yield a lipid to Laurdan ratio of 300:1. Emission maxima at 422 nm and 490 nm were recorded at the indicated temperatures and used to calculate the GP-values. Spectra normalization based on the Raman peak of H_2_O^56^ appearing at 389 nm for λ_EX_ 350 nm. (**b**) Determination of changes in DOPC membrane fluidity by Laurdan assay. Samples were treated for indicated durations with the kINPen source. **Δ**GP was calculated from Laurdan emission maxima at 422 nm and 490 nm. The change of fluidity is associated with the change of the GP value: a positive **Δ**GP indicates a decrease in fluidity and a negative **Δ**GP indicates an increase in fluidity.

To validate the simulation results, we investigated the influence of plasma-treatment on the fluidity of lipid bilayers as model membranes. DOPC liposomes were prepared with integration of the fluorescent probe 6-Dodecanoyl-2-dimethylaminonaphthalene (Laurdan). This lipophilic molecule inserts parallel to the lipid acyl chains and detects alterations in membrane fluidity as a consequence of a change in its dipole moment when water penetrates the membrane bilayer^46^. First, GP values were calculated for different temperatures (see Fig. 8a) using the emission maxima at 422 nm and 490 nm a (adapted from Parasassi *et al*.^55^):

$$GP=\frac{{I}_{422nm}-{I}_{490nm}}{{I}_{422nm}+\,{I}_{490nm}}$$

The determined GP value correlated inversely with temperature, and a change of + 1 °K resulted in a **Δ**GP of −0.0105, indicating that the model lipid fluidity increases with temperature. Experiments were performed in such way that no phase changes may occur, only changes in fluidity behavior of the disturbed lipid phase.

If temperature is kept constant, GP values can be used to assess lipid fluidity changes triggered by chemicals^47^. Time dependent impact of direct kINPen plasma treatment on the fluidity of DOPC liposomes was investigated and the corresponding change of the GP values (i.e., **Δ**GP) was detected. An immediate, treatment intensity dependent increase of GP in comparison to untreated DOPC liposomes occurred (see Fig. 8b). Afterwards, GP values decrease over time. While 5 and 10 min of treatment resulted in a negative **Δ**GP value already 30 min later, for 30 min plasma treatment the **Δ**GP was negative only after 2.5 h. After 3 h, all investigated samples had negative **Δ**GP, although the **Δ**GP of the 5 min plasma treated sample has decreased the most, indicating a higher water penetration, commonly interpreted as an increased fluidity in comparison to the untreated samples.

In summary, plasma treatment of DOPC liposomes revealed an initial and short-term higher rigidity of the lipid bilayer, but overall and in the long-term, a fluidization effect. This observation can be correlated to the simulation results, as ALD + OX2 leads to a higher disorder while OX2 alone leads to a lower disorder, but the effect of ALD + OX2 is more pronounced. So overall, our simulations predict a higher fluidity, assuming that the disorder correlates with fluidity. During or shortly after plasma treatment, a higher rigidity was observed. This is probably due to the initial OX2 oxidation. In a second step, presumably the ALD oxidation of lipid tails takes place, increasing the disorder. Indeed, the MS results showed that cross-linked structures are formed that are thought to be the consequence of the OX2 oxidation, which might allow water and ROS to penetrate deep into the lipid region from the sides of these cross-linked lipids, eventually causing lipid tail oxidation, i.e., ALD. Our long-term simulations already revealed that even OX2 and ALD oxidation occur together at the same time, and this overall leads to a decrease of the lipid order, thereby increasing the (cell membrane) fluidity.

Nevertheless, it has to be noted that the experiments intrinsically observe a different time scale than the calculated data. The experimental results will not be able to observe the short term results from the simulations. Although our simulations only show the head group and lipid tail oxidations, these will later on lead to more oxidations of other lipid tails, as a chain reaction (see the post plasma treatment times in Fig. 8b, e.g. after 1 hour). The effect of our simulations, although obtained for short time-scales, can thus be correlated to a longer-term effect, i.e., after plasma treatment, in the experiments. It should also be mentioned that according to the density profiles of ROS across the PLB obtained by Cordeiro^13^, the OH radicals preferably stay closer to the carbonylester groups, where OX2 can take place. As mentioned above, this might then explain the higher ordering seen in the experiments upon direct plasma treatment. The other oxidation product obtained from the reactive simulations (i.e., OX1) was not observed by the MS measurements, so it might in reality not occur, as the results were obtained in vacuum, i.e., without taking into account the stabilizing effect of water covering the head group; this was done due to the severe computational cost of DFTB. Indeed, in vacuum, the polar groups (i.e., choline and phosphate) are much more pronounced and the choline group can attract the OH radical, which can influence the reaction statistics, while in a “real” structure with a stabilizing water layer, the influence of the polar groups to the statistics will be lower.

We should also mention here that the simulation results presented in this study are the effects of primary (or single) impacts of OH radicals on the structure. In reality, a consecutive number of OH radicals can impinge the PLB and react with the byproducts created from primary interactions, leading to the further destruction of the system. Moreover, plasma is not solely a source of OH radicals but also of other ROS as well as charged species. Future work is thus needed to further correlate experiments with the simulations.

In summary, although the experiments and simulations cannot be directly compared due to the vastly different time scales, they certainly provide a complementary view. Moreover, they both illustrate that ROS from plasma treatment affect the ordering and fluidity of the PLB, in some cases leading to higher ordering (or lower fluidity) and in other cases to lower ordering (and higher fluidity).

## Conclusions

We employed different modeling approaches, by a combination of reactive (DFTB) and non-reactive MD as well as DFT, to study the interaction of ROS (namely OH, HO_2_ and H_2_O_2_) with the head groups of the PLB, as well as the subsequent longer-term behavior of the oxidized PLB at a time-scale of 100 ns. We used POPC and DOPC bilayers as model systems and found the same trends for both.

The aim of our study was to elucidate at the atomic scale (a) which of the ROS can react with the head groups and possibly destroy them, and (b) what is the consequence of the oxidation of the head groups, as well as the lipid tails, on the structural and dynamic properties of the PLB. We focused on the interaction with the head groups, as they will first be attacked by the ROS, and the structural changes of the PLB as a result of the head group oxidations might open a path for the later lipid tail oxidation. Therefore, this study can elucidate the onset of lipid (per)oxidation.

Our reactive (DFTB) MD simulations showed that the aforementioned ROS are in general able to penetrate through the water layer and eventually reach the head groups of the PLB. However, only the OH radicals were found to react with the head groups, leading to the dissociation of some bonds. The results show that the reactions of OH radicals are always initiated by the abstraction of a H atom from (different parts of) the head groups. Two oxidation mechanisms (i.e., OX1 and OX2) seemed to lead to the destruction of the head groups and the detachment of some molecules.

In parallel, MS measurements were performed in order to validate the reactive (DFTB) MD simulations, as well as to check the effect of lipid tail composition on the properties of the oxidized PLBs. Analysis of our MS data showed that OX2 and ALD oxidation products are more prominent than OX1. Therefore, in our further (longer time-scale) simulations we applied only these two oxidation products.

The longer-term behavior of the oxidized bilayer was studied using the non-reactive MD simulations. The simulations predicted that the OX2 oxidation product yields a decrease in the area per lipid, an increase in the bilayer thickness and a higher ordering of the lipid tails, which eventually leads to a drop of the cell membrane fluidity. The consequence of OX2 is mainly due to the stiffening effect of the detached lipid tails, similar to the effect of cholesterol^22^, which makes the bilayer slightly more rigid and less fluidic. On the other hand, the results of the combined effect of OX2 together with the ALD showed that if these two oxidations take place together, this overall yields an increase in the area per lipid, a drop in the bilayer thickness, as well as a lower ordering of the lipid tails, which eventually leads to an increase of the cell membrane fluidity. This opposite effect of the combined oxidation (i.e., OX2 and ALD) is mainly due to the detached short aldehyde tails that can freely move towards the head groups ﻿as well as the shortened tails which bend towards the head groups, thereby increasing the area per lipid, which ultimately leads to an increase in PLB fluidity.

The results of the non-reactive MD simulations were also validated by comparison with the experiments, where the change in membrane fluidity of DOPC liposomes upon plasma treatment was assessed using the fluorescent probe Laurdan. The modifications on the PLB by reactive species originating from the plasma lead eventually to an ongoing fluidization of the membrane, though it is preceded by a slight and time-limited increase in rigidity. These modifications continue beyond the termination of the plasma treatment and thus indicate a chain reaction leading to a lasting pronounced disorder in membrane lipid packing.

Our study is particularly interesting for cancer treatment by therapies which produce (extracellular) ROS, such as plasma treatment, but also e.g., chemo- and radiation therapy. Indeed, these ROS will interact with the plasma membrane, and in first instance with the head groups of the PLB. Our simulations revealed that these interactions cause oxidation of the head groups followed by lipid tail oxidation, eventually leading to an increase in the cell membrane fluidity. This might then allow the ROS to penetrate further through the membrane into the cell interior (e.g., through the pores formed after the oxidation of the lipid tails) and cause intracellular oxidative damage, eventually leading to cell death.

It should be realized that cell death is a complex event, involving not only the PLB but also signaling proteins. Therefore, it is also important to study how oxidation impacts on these biomolecules. This will be the subject of our future investigation. Nevertheless, we believe that our present results already provide some atomic level insights into the mechanisms of ROS interacting with the head groups of the PLB and that they can elucidate the onset of the lipid (per)oxidation process.

## Electronic supplementary material

Supplementary Information

## References

[1] Schlegel J, Köritzer J, Boxhammer V (2013). Plasma in cancer treatment. Clinical Plasma Medicine.

[2] Keidar M (2011). Cold plasma selectivity and the possibility of a paradigm shift in cancer therapy. British journal of cancer.

[3] Ratovitski EA (2014). Anti-Cancer Therapies of 21st Century: Novel Approach to Treat Human Cancers Using Cold Atmospheric Plasma. Plasma Processes and Polymers.

[4] Graves DB (2014). Reactive Species from Cold Atmospheric Plasma: Implications for Cancer Therapy. Plasma Processes and Polymers.

[5] Köritzer J (2013). Restoration of Sensitivity in Chemo—Resistant Glioma Cells by Cold Atmospheric Plasma. PLoS one.

[6] Brullé L (2012). Effects of a non thermal plasma treatment alone or in combination with gemcitabine in a MIA PaCa2-luc orthotopic pancreatic carcinoma model. PloS one.

[7] Lu X (2016). Reactive species in non-equilibrium atmospheric-pressure plasmas: Generation, transport, and biological effects. Physics Reports.

[8] Ishaq M, Evans MM, Ostrikov KK (2014). Effect of atmospheric gas plasmas on cancer cell signaling. International Journal of Cancer.

[9] Valko M, Rhodes C, Moncol J, Izakovic M, Mazur M (2006). Free radicals, metals and antioxidants in oxidative stress-induced cancer. Chemico-biological interactions.

[10] Trachootham D, Alexandre J, Huang P (2009). Targeting cancer cells by ROS-mediated mechanisms: a radical therapeutic approach?. Nature reviews Drug discovery.

[11] Kaneko T (2015). Improvement of cell membrane permeability using a cell-solution electrode for generating atmospheric-pressure plasma. Biointerphases.

[12] Hong S-H, Szili EJ, Jenkins ATA, Short RD (2014). Ionized gas (plasma) delivery of reactive oxygen species (ROS) into artificial cells. Journal of Physics D: Applied Physics.

[13] Cordeiro RM (2014). Reactive oxygen species at phospholipid bilayers: distribution, mobility and permeation. Biochimica et biophysica acta.

[14] Moller M, Lancaster JR, Denicola A (2008). The interaction of reactive oxygen and nitrogen species with membranes. Curr Top Membr.

[15] Tai W-Y (2010). Interplay between structure and fluidity of model lipid membranes under oxidative attack. The Journal of Physical Chemistry B.

[16] Grzelinska E, Bartosz G, Gwozdzinski K, Leyko W (1979). A spin-label study of the effect of gamma radiation on erythrocyte membrane. Influence of lipid peroxidation on membrane structure. International Journal of Radiation Biology.

[17] Wratten ML (1992). Structural and dynamic effects of oxidatively modified phospholipids in unsaturated lipid membranes. Biochemistry.

[18] Chen JJ, Yu BP (1994). Alterations in mitochondrial membrane fluidity by lipid peroxidation products. Free Radical Biology and Medicine.

[19] Mason RP, Walter MF, Mason PE (1997). Effect of oxidative stress on membrane structure: small-angle X-ray diffraction analysis. Free Radical Biology and Medicine.

[20] Wong-Ekkabut J (2007). Effect of lipid peroxidation on the properties of lipid bilayers: a molecular dynamics study. Biophysical journal.

[21] Beranova L, Cwiklik L, Jurkiewicz P, Hof M, Jungwirth P (2010). Oxidation changes physical properties of phospholipid bilayers: fluorescence spectroscopy and molecular simulations. Langmuir.

[22] Van der Paal J, Neyts EC, Verlackt CC, Bogaerts A (2016). Effect of lipid peroxidation on membrane permeability of cancer and normal cells subjected to oxidative stress. Chemical Science.

[23] Yusupov M, der Paal VJ, Neyts E, Bogaerts A (2017). Synergistic effect of electric field and lipid oxidation on the permeability of cell membranes. Biochimica et Biophysica Acta (BBA)-General Subjects.

[24] Richter C (1987). Biophysical consequences of lipid peroxidation in membranes. Chemistry and physics of lipids.

[25] Niki E, Yoshida Y, Saito Y, Noguchi N (2005). Lipid peroxidation: mechanisms, inhibition, and biological effects. Biochemical and biophysical research communications.

[26] Cwiklik L, Jungwirth P (2010). Massive oxidation of phospholipid membranes leads to pore creation and bilayer disintegration. Chemical Physics Letters.

[27] Alberts, B. *et al*. Molecular Biology of the Cell. New York: Garland Science; 2008. *Classic textbook now in its 5th Edition* (2010).

[28] Elstner M (1998). Self-consistent-charge density-functional tight-binding method for simulations of complex materials properties. Physical Review B.

[29] Gaus M, Goez A, Elstner M (2012). Parametrization and benchmark of DFTB3 for organic molecules. Journal of Chemical Theory and Computation.

[30] Neyts EC, Yusupov M, Verlackt CC, Bogaerts A (2014). Computer simulations of plasma–biomolecule and plasma–tissue interactions for a better insight in plasma medicine. Journal of Physics D: Applied Physics.

[31] Bogaerts, A. *et al*. Multi-level molecular modeling for plasma medicine. *Journal of Physics D: Applied Physics* **49** 054002 (2016).

[32] Chiu S-W, Pandit SA, Scott H, Jakobsson E (2009). An improved united atom force field for simulation of mixed lipid bilayers. The Journal of Physical Chemistry B.

[33] Dünnbier M (2013). Ambient air particle transport into the effluent of a cold atmospheric-pressure argon plasma jet investigated by molecular beam mass spectrometry. J Phys D Appl Phys.

[34] Metelmann H-R (2012). Experimental Recovery of CO2-Laser Skin Lesions by Plasma Stimulation. Am J. Cosmetic Surg..

[35] von Woedtke T, Metelmann HR, Weltmann KD (2014). Clinical Plasma Medicine: State and Perspectives of *in Vivo* Application of Cold Atmospheric Plasma. Contributions to Plasma Physics.

[36] Kramer A (2013). Suitability of tissue tolerable plasmas (TTP) for the management of chronic wounds. Clinical Plasma Medicine.

[37] Partecke LI (2012). Tissue tolerable plasma (TTP) induces apoptosis in pancreatic cancer cells *in vitro* and *in vivo*. BMC Cancer.

[38] Metelmann H-R (2015). Head and neck cancer treatment and physical plasma. Clinical Plasma Medicine.

[39] Winter J (2014). Tracking plasma generated H2O2from gas into liquid phase and revealing its dominant impact on human skin cells. Journal of Physics D: Applied Physics.

[40] Schmidt-Bleker A, Winter J, Bösel A, Reuter S, Weltmann K-D (2016). On the plasma chemistry of a cold atmospheric argon plasma jet with shielding gas device. Plasma Sources Science and Technology.

[41] Reuter S (2015). The Influence of Feed Gas Humidity Versus Ambient Humidity on Atmospheric Pressure Plasma Jet-Effluent Chemistry and Skin Cell Viability. IEEE Transactions on Plasma Science.

[42] Reuter S (2012). From RONS to ROS: Tailoring Plasma Jet Treatment of Skin Cells. Ieee Transactions on Plasma Science.

[43] Jablonowski H (2015). Impact of plasma jet VUV-radiation on Reactive Oxygen Species Generation in Bio-Relevant Liquids. Physics of Plasmas.

[44] Winter J (2013). Feed gas humidity: a vital parameter affecting a cold atmospheric-pressure plasma jet and plasma-treated human skin cells. J Phys D Appl Phys.

[45] Reuter S (2012). Controlling the Ambient Air Affected Reactive Species Composition in the Effluent of an Argon Plasma Jet. IEEE Transactions on Plasma Science.

[46] Chong PL-G, Wong PTT (1993). Interactions of Laurdan with phosphatidylcholine liposomes: a high pressure FTIR study. Biochimica et Biophysica Acta (BBA) - Biomembranes.

[47] Parasassi T, De Stasio G, Ravagnan G, Rusch RM, Gratton E (1991). Quantitation of lipid phases in phospholipid vesicles by the generalized polarization of Laurdan fluorescence. Biophysical journal.

[48] Yusupov M (2014). Reactive molecular dynamics simulations of oxygen species in a liquid water layer of interest for plasma medicine. Journal of Physics D: Applied Physics.

[49] Chalmet S, Ruiz-Lopez MF (2006). The structures of ozone and HOx radicals in aqueous solution from combined quantum/classical molecular dynamics simulations. J Chem Phys.

[50] Moin ST, Hofer TS, Randolf BR, Rode BM (2012). An ab initio quantum mechanical charge field molecular dynamics simulation of hydrogen peroxide in water. Computational and Theoretical Chemistry.

[51] Verlackt C, Neyts E, Bogaerts A (2017). Atomic scale behavior of oxygen-based radicals in water. Journal of Physics D: Applied Physics.

[52] van Blitterswijk WJ, Verheij M (2013). Anticancer mechanisms and clinical application of alkylphospholipids. Biochimica et Biophysica Acta (BBA)-Molecular and Cell Biology of Lipids.

[53] Vink SR, van Blitterswijk WJ, Schellens JH, Verheij M (2007). Rationale and clinical application of alkylphospholipid analogues in combination with radiotherapy. Cancer treatment reviews.

[54] Dorlo TP, Balasegaram M, Beijnen JH, de Vries PJ (2012). Miltefosine: a review of its pharmacology and therapeutic efficacy in the treatment of leishmaniasis. Journal of Antimicrobial Chemotherapy.

[55] Parasassi T, De Stasio G, d’Ubaldo A, Gratton E (1990). Phase fluctuation in phospholipid membranes revealed by Laurdan fluorescence. Biophysical journal.

[56] Lawaetz AJ, Stedmon CA (2009). Fluorescence intensity calibration using the Raman scatter peak of water. Appl Spectrosc.

