# Supplementary material for: Effect of head group and lipid tail oxidation in the cell membrane revealed through integrated simulations and experiments
Source: Sci Rep. 2017 Jul 18;7:5761. doi: 10.1038/s41598-017-06412-8 (PMC5515852; doi:10.1038/s41598-017-06412-8)
Supplement: Supplementary file 1 — Supplementary Information [file 41598_2017_6412_MOESM1_ESM.pdf]

Supplementary Information

Effect of head group and lipid tail oxidation in the cell membrane revealed through integrated simulations and experiments

M Yusupov[1], K Wende[2], S Kupsch[3], E C Neyts[1], S Reuter[2] and A Bogaerts[1]

[1] Research Group PLASMANT, Department of Chemistry, University of Antwerp, Universiteitsplein 1, B-2610 Antwerp, Belgium
[2] Leibniz Institute for Plasma Science and Technology, INP Greifswald e.V. Felix-Hausdorff-Str. 2, 17489 Greifswald, Germany
[3] Leibniz-Center for Medicine and Biosciences, Research Center Borstel, Division of Immunobiophysics, Parkallee 1-40, 23845 Borstel, Germany

E-mail: maksudbek.yusupov@uantwerpen.be

Simulation details

Reactive (DFTB) MD simulations

In our reactive MD simulations we use the so-called DFTB3 method, which is the extended and improved version of self-consistent charge DFTB [1]. It is derived from a third order expansion of the DFT total energy expression, and accurately describes the H binding energies, proton affinities and H transfer barriers without losing its speed and robustness [1]. In this work, we use a parameter set (so called "3OB", emphasizing the use for Organic and Bio-molecules) that was specifically developed for DFTB3 [2], containing parameters for C/H/O/N/S/P atoms.

The model system for the DFTB simulations consists of 8 DOPC (or POPC) molecules with water layers on top and at the bottom (see Fig. 1b in the main paper) that are placed in a box with dimensions of ~16 Å × 72 Å × 18 Å. The geometry of the structure is optimized using the conjugate gradient algorithm. The system is then equilibrated for 5 ps (i.e., 10^4 iterations) in the isothermal-isobaric (i.e., NPT) ensemble at 300 K, employing the Berendsen thermostat and barostat [3] with a coupling constant of 100 fs and 700 fs, respectively. In all simulations (i.e., during the thermalization, as well as during the particle impact simulations) we use a time step of 0.5 fs. Periodic boundary conditions are applied in all three directions to represent a bulk system.

The impinging plasma species first interact with the water layer before reaching the head group of the PLB and therefore to study their behavior in the water layer we perform some test runs by creating a single plasma species in a small box of water (including 33 water molecules with a density of 1 g/cm^3) with dimensions of 10 Å × 10 Å × 10 Å. The water structure (with the single particle in it) is optimized as explained above and then equilibrated in the isothermal (i.e., NVT) ensemble at 300 K, employing the Berendsen thermostat [3] with a coupling constant of 100 fs.

The model system consisting of a single PL molecule (which contains 54 atoms) is placed in a box with size of 25 Å × 25 Å × 25 Å. Note that the volume of the box is much larger than the system itself. This is done to randomly create the plasma particle around the structure. Again this system is optimized using the conjugate gradient algorithm and equilibrated for 10 ps in the NVT ensemble. Subsequently, a single plasma species (e.g., an OH radical) is randomly created around the structure with a minimum distance of 5 Å from the system. We perform 100 simulations for each impinging species, i.e., for OH, HO_2 and H_2O_2, and each simulation lasts for 15 ps (i.e., 3×10^4 iterations), which is long enough to observe the bond breaking/formation processes.

For the analysis of the possible reactions that are obtained from the single PL molecule we use the "real" water-stabilized PLB structure (i.e., the structure with 8 PLs, presented in Fig. 1b of the main paper). Because of the high computational cost of the DFTB method, we place the species closer (at ~2 Å) to the position where they should react, based on the results obtained for the single PL molecule. Subsequently, we

perform again MD simulations, now for 5 ps (i.e., 10^4 iterations), because of the high computational cost of the DFTB method. However, since the plasma species are positioned close enough to the place where they should react, this time is already sufficient for the reaction to occur.

Non-reactive MD simulations

In our non-reactive MD simulations the model systems with OX2 oxidation products are created from the non-oxidized (i.e., native) PLBs containing 128 PLs, by replacing 12, 32, 64 and 128 DOPC (or POPC) molecules with OX2, corresponding to concentrations of 9.4%, 25%, 50% and 100%, respectively. In the case of ALD oxidation products, we keep 25% of the OX2 products and replace the other intact DOPC molecules with 16, 32, 64 and 96 of ALD products, the concentrations of which correspond to 12.5%, 25%, 50% and 75%, respectively. Thus, in the fully oxidized PLB we will have either 100% of OX2 or 25% of OX2 and 75% of ALD oxidation products. Note that the two bilayer leaflets always contain the same number of oxidized PLs in our model systems. In this way, we obtain 9 different bilayer systems (including the intact PLB) for DOPC and 5 for POPC. All 14 model systems are created using the Packmol package [4]. Each system is then optimized using the steepest descent algorithm and equilibrated for 80 ns in the NPT ensemble, at 300 K and 1 atmosphere, employing the Nosé-Hoover thermostat with a coupling constant of 0.2 ps [5] as well as the semi-isotropic Parinello-Rahman barostat with a compressibility and coupling constant of $4.5\text{x}10^{-5}$ bar^{-1} and 1 ps, respectively [6]. Subsequently, MD simulations are run for 100 ns applying again the NPT ensemble. In all simulations a time step of 2 fs is used. Periodic boundary conditions are applied in all directions. The simulations are carried out using GROMACS 4.6 software, applying the classical united-atom GROMOS 43A1-S3 force field [7], which contains parameters for a wide variety of lipids, including DOPC and POPC. Table S1 shows the comparison for the average surface area per lipid and the bilayer thickness of the intact PLB, between our calculated values and the values obtained from experiments and other computational studies. It is clear that our computational results are well within the range of data reported in literature [8-16].

<u>Table S1.</u> Comparison between our calculated data and experimental or other computational data from literature, for pure DOPC and POPC bilayers.

phospholipid	Properties	Calculated value	Data from literature
DOPC	Surface area per lipid (nm^2)	0.644 ± 0.001	0.62 – 0.72
	Bilayer thickness (nm)	3.902 ± 0.007	3.67 – 4.0
POPC	Surface area per lipid (nm^2)	0.639 ± 0.001	0.63 – 0.643
	Bilayer thickness (nm)	3.782 ± 0.012	3.75 – 3.91

DFT simulations

We also performed DFT calculations to obtain new parameters for the non-reactive force field (for the oxidized/modified bilayers), by carrying out a fitting of some standard potential energy functions to the DFT energy. For this purpose we used the ADF modeling suite package [17,18], employing the B3LYP functional [19] with the Slater-type basis set TZ2P [20]. The implemented new parameters to the force field are given in Table S2. For the calculation of bond and angle (as well as improper dihedral) force constants, we restrained the bond lengths and angles at seven different values and then fitted a harmonic potential function to the energy profile. For the proper dihedral parameters, dihedral angles were restrained at 36 different values from 0 to 360°, and the corresponding dihedral function was fitted to the potential energy (see Table S2). Note that we performed the parametrization (or fitting) procedure only for those bonds or angles or dihedrals which are not included in the force field. Moreover, we used only those Lennard-Jones parameters which are present in the force field.

Table S2. New parameters used in the GROMOS 43A1-S3 force field for describing the newly formed bonds, angles and dihedrals in the oxidized PLBs. The functional forms used for the bonds, angles and dihedrals can be found in the manual of GROMACS 4.6 [21]*. Note that C atoms given in the table are united atoms (see Fig. S1).*

**Bonds**			
Bond	b_0 (nm)	k_b (kJ.mol^{-1}.nm^{-4})	functional type (see [21])
C_1-N_4 (Fig. S1a)	0.148	5.5×10^6	2
C_3-N_4 (Fig. S1a)	0.148	5.5×10^6	2
C_2-N_4 (Fig. S1a)	0.127	2.03×10^7	2
**Angles**			
Angle	θ_0 (deg)	k_θ (kJ.mol^{-1})	functional type (see [21])
C_1-N_4-C_2 (Fig. S1a)	122.2	1257.42	2
C_3-N_4-C_2 (Fig. S1a)	122.2	1257.42	2
C_1-N_4-C_3 (Fig. S1a)	115.6	1020.91	2
H_1-O_2-C_3 (Fig. S1b)	104.8	498.76	2
O_1-C_2-C_3 (Fig. S1c)	123.1	1144.56	2
H_1-O_2-C_3 (Fig. S1d)	108.8	962.27	2
**Dihedral angles**			
improper dihedral	ξ_0 (deg)	k_ξ (kJ.mol^{-1}.rad^{-2})	functional type (see [21])
N_4-C_1-C_3-C_2 (Fig. S1a)	0.0	618.37	2
N_4-C_2-C_1-C_3 (Fig. S1a)	0.0	436.23	2
N_4-C_2-C_3-C_1 (Fig. S1a)	0.0	436.23	2
proper dihedral (multiple)	ϕ_s (deg)	k_ϕ (kJ.mol^{-1})	functional type and multiplicity (see [21])
H_1-O_2-C_3-C_4 (Fig. S1b)	227.36	13.59	9 ; 1
H_1-O_2-C_3-C_4 (Fig. S1b)	265.06	2.78	9 ; 2
H_1-O_2-C_3-C_4 (Fig. S1b)	341.71	2.94	9 ; 3
P-O_1-C_2-C_3 (Fig. S1c)	5.33	7.17	9 ; 1
P-O_1-C_2-C_3 (Fig. S1c)	194.14	6.89	9 ; 2
proper dihedral (Ryckaert-Bellemans)	C_0, C_1, C_2, C_3, C_4, C_5 (kJ.mol^{-1})		functional type (see [21])
O_2-C_3-C_4-O_5 (Fig. S1b)	16.8601; -35.2383; -7.4278; 38.6893; 12.4651; -3.4948		3
C_2-C_3-C_4-O_5 (Fig. S1c)	3.91271; 15.5029; 5.7707; -16.09216; 0.36505; -3.90425		3
H_1-O_2-C_3-C_4 (Fig. S1d)	1.68792; -7.08289; 2.94293; 11.0719; -1.76568; -1.48307		3
O_2-C_3-C_4-C_5 (Fig. S1d)	6.39811; -22.80372; 3.16939; 35.85167; 1.22503; -1.93274		3
**Partial charges**			
atom	charge	atom	charge
C_1 (Fig. S1a)	0.3	C_2 (Fig. S1c)	0.2
C_2 (Fig. S1a)	0.4	C_3 (Fig. S1c)	0.0
C_3 (Fig. S1a)	0.3	C_4 (Fig. S1c)	0.3
N_4 (Fig. S1a)	0.0	O_5 (Fig. S1c)	-0.4
H_1 (Fig. S1b)	0.3	C_6 (Fig. S1c)	0.5
O_2 (Fig. S1b)	-0.6	O_7 (Fig. S1c)	-0.5
C_3 (Fig. S1b)	0.3	H_1 (Fig. S1d)	0.3
C_4 (Fig. S1b)	0.3	O_2 (Fig. S1d)	-0.6
O_5 (Fig. S1b)	-0.6	C_3 (Fig. S1d)	0.3
P_6 (Fig. S1b)	1.7	C_4 (Fig. S1d)	0.0
O_7 (Fig. S1b)	-0.9	C_5 (Fig. S1d)	0.0
O_8 (Fig. S1b)	-0.9		
O_9 (Fig. S1b)	-0.6		

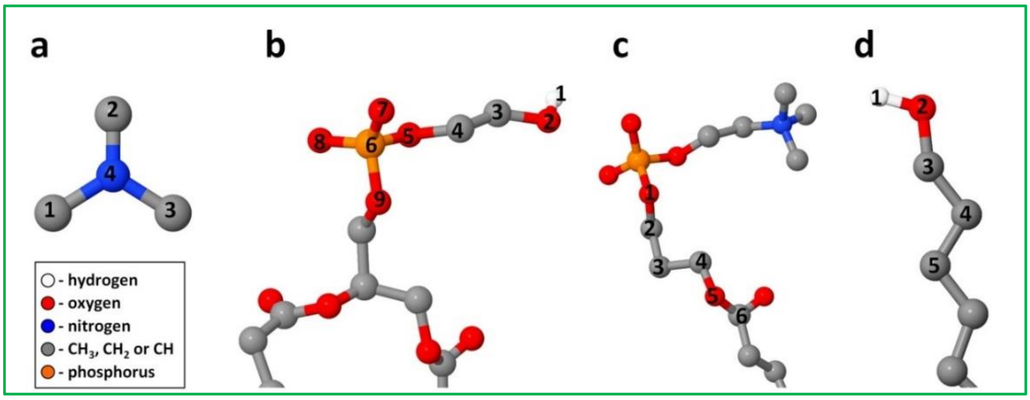

Figure S1: *Schematic picture of the fragments of an oxidized (modified) DOPC (or POPC) PLB for OX1 ((a) and (b)) as well as for OX2 ((c) and (d)), cf. Fig. 5b,c in the main paper.*

It should be mentioned here that initially we performed the parametrization for both OX1 and OX2 oxidation products, since our reactive (DFTB) MD simulation results showed these two oxidation products to be most abundantly formed. However, the experimental results did not reveal OX1 as a prominent oxidation product, and therefore we did not use the parameters of this oxidation product in our further non-reactive MD simulations.

Subsequent reaction of a new OH radical

Figure S2 illustrates the reaction energies of the two possible subsequent reactions of the head group oxidized with OX1, with a new OH radical (see also Fig. 2 in the main paper). A new OH radical can either abstract the H atom from the CH_2 group (see initial structure in the red curve of Fig. S2), forming a stable double C=C bond and water molecule (see final structure in the red curve) or it can react with the C radical (see initial structure in the black curve), forming an alcohol group (see final structure in the black curve). As is clear from Fig. S2, the calculated reaction energies employing the above described DFT method revealed that the reaction in which the alcohol group is formed (see final structure in the black curve) is energetically slightly more favorable (cf. ΔE_2 in Fig. S2). Moreover, both reactions do not have a barrier. Note that to calculate the reaction energies of these two systems we performed a constrained optimization at 25 different points. In other words, we restrained the distance between an O atom of the OH radical and an H atom of the CH_2 residue connected to the phosphate group (see initial structure in red curve) and the distance between the O atom of the OH radical and the C radical (see initial configuration in black curve), at 25 different values and calculated the energy profiles.

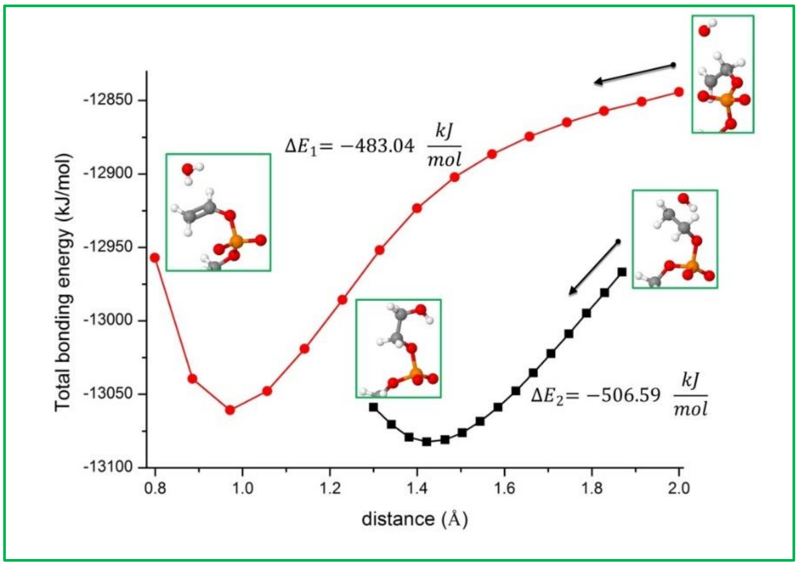

Figure S2: *Reaction energies of two systems. The calculated reaction energies show that the reaction presented by the black curve (i.e., the reaction in which a new OH group is created, instead of a double C=C bond and a water molecule) is energetically more favorable.*

Reactive (DFTB) MD results

As mentioned above, the reactions of the OH radicals with the head group of the PL were studied using reactive (DFTB) MD simulations. To get some limited statistics we performed 100 MD simulations. According to the simulation results, we observed six reaction mechanisms which are presented in Fig. S3. For clarity, we named these mechanisms as OX1, OX2, …, OX6. As is clear from Fig. S3, all of these reaction mechanisms are initiated by H-abstraction from (different parts of) the head groups, but few of them give rise to further bond breaking. Note that the OX6 reaction mechanism can take place in both lipid tails (beneath the ester groups, see Fig. S3) and the percentage given is the sum of the OX6 occurrences in both tails. A similar summation over the CH_3 groups is also applied for OX1. We did not investigate the further reaction of a new OH radical with the modified PL (by OX1–X6) due to the computational cost of the DFTB method, except for OX1, where we performed further DFT calculations (see above) to find out the energetically most favorable reaction product. It turned out that the formation of an alcohol group was most favorable, therefore we assumed the same for the other modified PLs. This is indeed the most logical for these structures.

We studied the longer-term behavior of only OX2 (as well as ALD, see the main text) in our non-reactive MD simulations and we did not study the consequences of the other mechanisms. The reason is that the experimental results suggested only OX2 together with ALD to be the most prominent oxidation products.

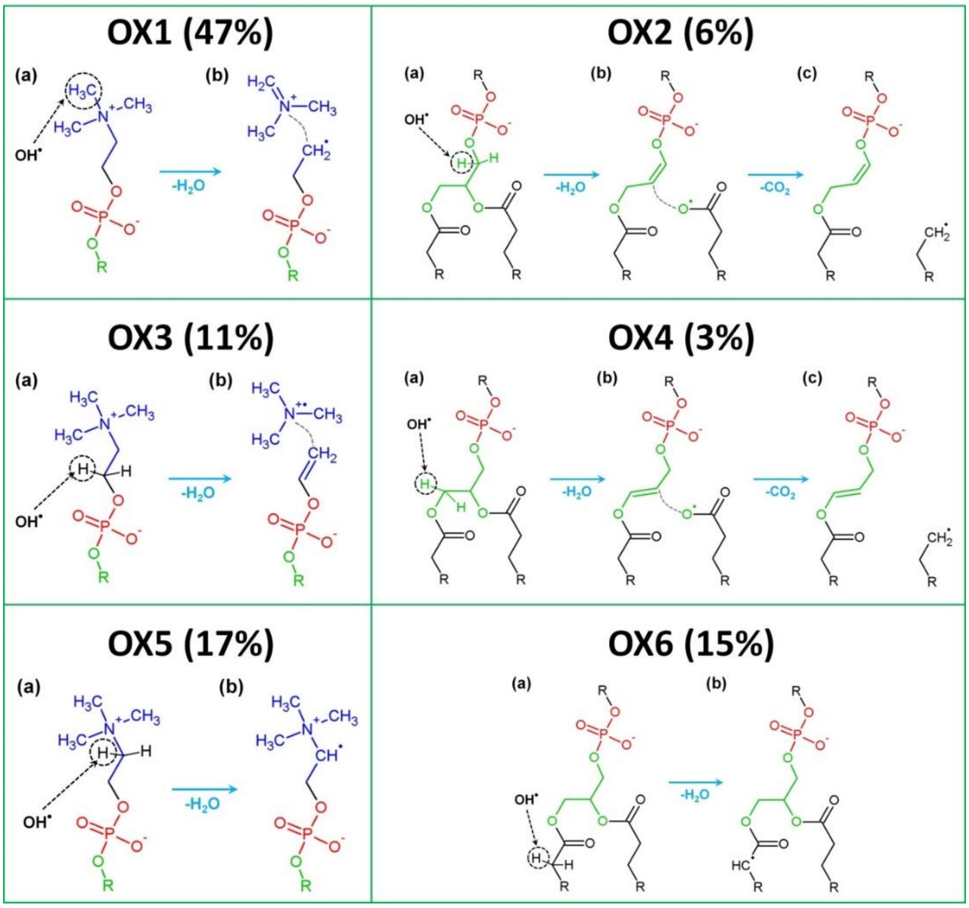

Figure S3: *Schematic illustration of six (OX1 to OX6) reaction mechanisms observed in the reactive (DFTB) MD simulations, with their relative occurrences in brackets. The consequences of OX3 and OX4 are similar to the consequences of OX1 and OX2, respectively, whereas OX5 and OX6 do not lead to the destruction of the head group and are therefore unimportant for the longer term simulations.*

Non-reactive MD results

The surface area per lipid and the bilayer thickness are plotted in Fig. S4 as a function of the concentration of the oxidized PLs, for the OX2 reaction mechanism on the POPC and DPPC PLBs. As is

clear, similar trends were obtained for the POPC and DPPC bilayers as for the DOPC PLB (see section Non-reactive MD results in the main paper for a detailed explanation). The only difference is that the relative changes (increase and decrease) in the values are more pronounced in the case of DPPC (see Fig. S4c,d). This is probably due to the difference in the melting temperature of the different PLs. At the simulated temperature (i.e., 300 K), DPPC is in the gel phase ($T_m \approx 328$ [22]), while POPC and DOPC ($T_m \approx 294$ K and 274 K, respectively [22]) are already in the liquid state (i.e., with more disordered lipids). Detachment of a lipid tail (see Fig. 5c in the main paper) has a stiffening effect on the PLB, which makes that the OX2 oxidized DPPC bilayers remain in the gel phase. Finally, it is clear from Fig. S4c,d that both the area per lipid and the bilayer thickness level off after 50% oxidation with OX2, which indicates that the lipids are already nearly perfectly structured in the DPPC system. Hence, both the area per lipid as well as the bilayer thickness cannot further decrease/increase.

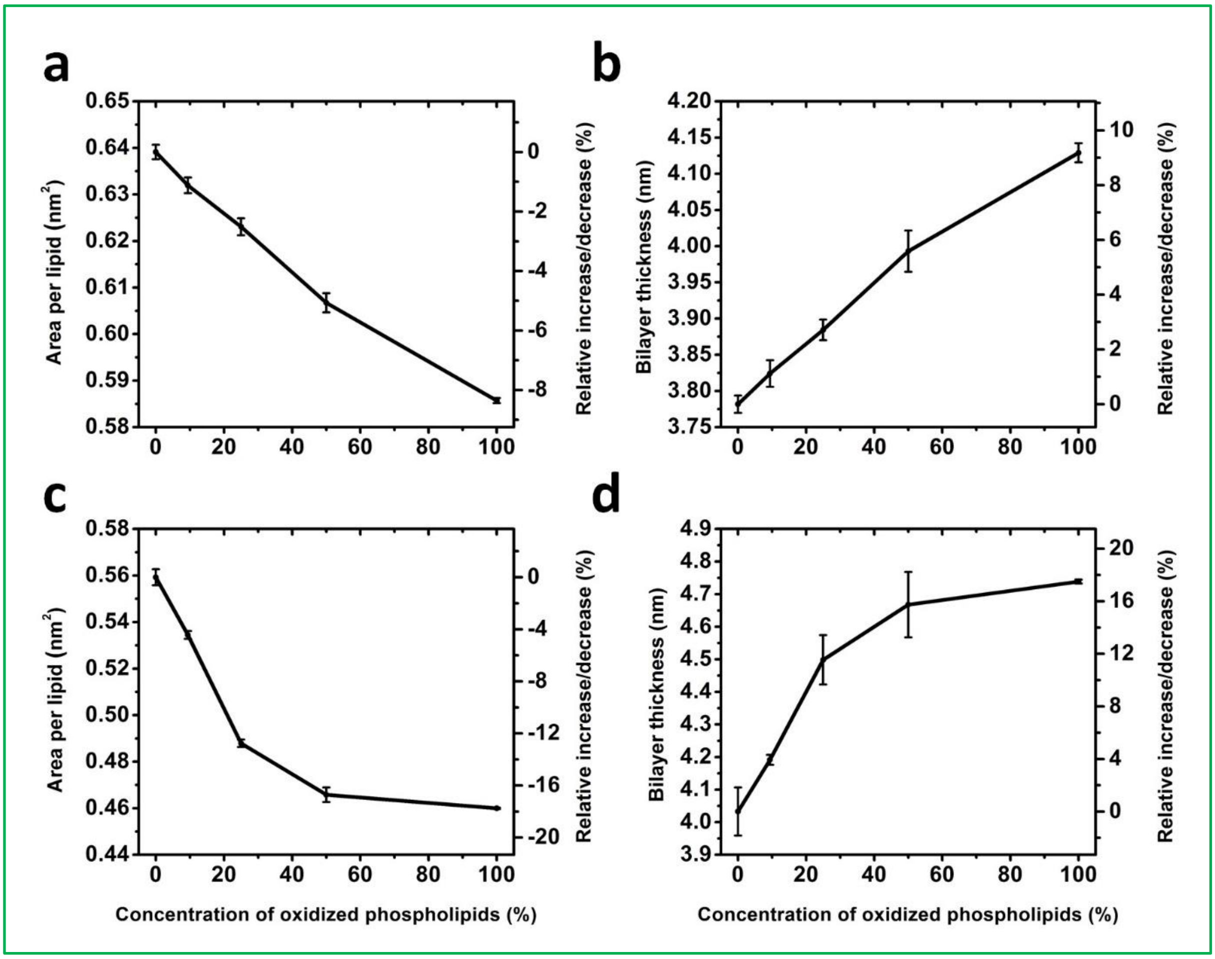

<u>Figure S4:</u> Surface area per lipid and thickness of the bilayer as a function of the concentration of the oxidized PLs, for the OX2 oxidation product, for the POPC ((a) and (b)) and DPPC ((c) and (d)) PLBs.

Results of high resolution mass spectrometry

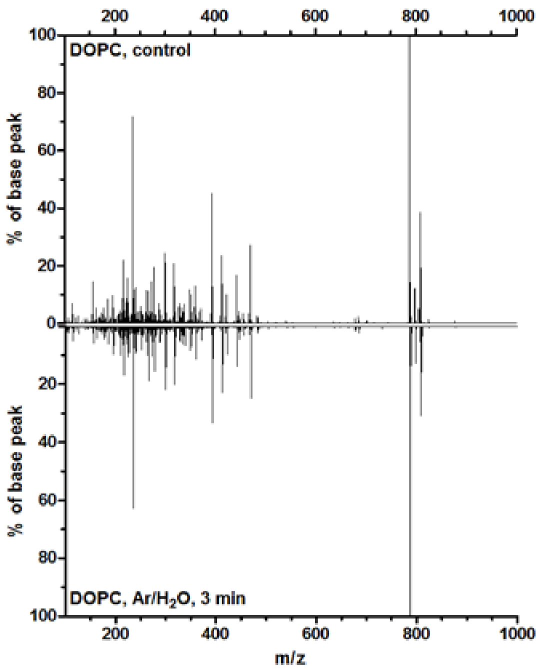

Figure S5: Mirror plot of DOPC control (top) and plasma treated DOPC (bottom). Data generated from direct infusion, electrospray ionization / TripleTOF5600 high resolution mass spectrometry. Data normalized to base peak count (DOPC, ≈ 20,000/s). Only minor differences were detected between treated and untreated DOPC (see text). Peaks with higher m/z belong to alkali and earth alkali adducts of DOPC. In the low m/z range in source fragments and impurities/traces from buffers occur.

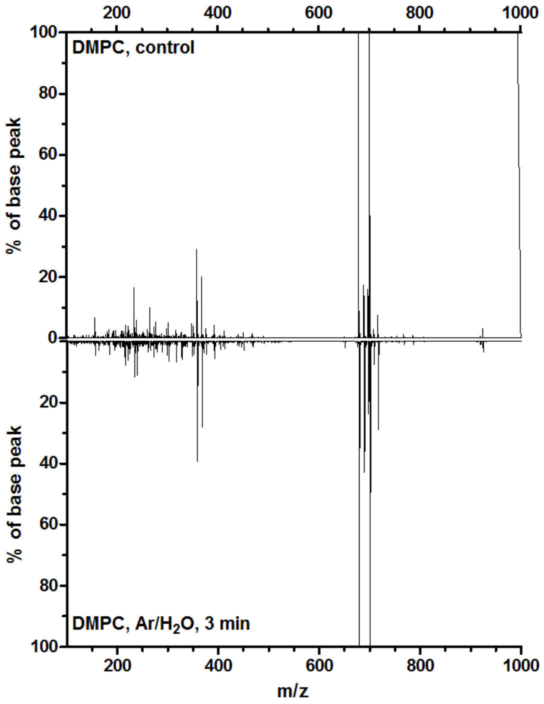

Figure S6: Mirror plot of DMPC control (top) and plasma treated DMPC (bottom). Data generated from direct infusion, electrospray ionization / TripleTOF5600 high resolution mass spectrometer. Data normalized to base peak count (DMPC, ≈ 40,000/s). Only minor differences were detected between treated and untreated DMPC (see text). Peaks with higher m/z belong to alkali and earth alkali adducts of DMPC. In low m/z range, major peaks belong to doubly charged ions of DMPC + magnesium or calcium.

References

1 Gaus, M., Goez, A. & Elstner, M. Parametrization and benchmark of DFTB3 for organic molecules. *Journal of Chemical Theory and Computation* **9**, 338-354 (2012).

2 Gaus, M., Lu, X., Elstner, M. & Cui, Q. Parameterization of DFTB3/3OB for Sulfur and Phosphorus for chemical and biological applications. *Journal of chemical theory and computation* **10**, 1518-1537 (2014).

3 Berendsen, H. J., Postma, J. P. M., van Gunsteren, W. F., DiNola, A. & Haak, J. Molecular dynamics with coupling to an external bath. *The Journal of chemical physics* **81**, 3684-3690 (1984).

4 Martínez, L., Andrade, R., Birgin, E. G. & Martínez, J. M. PACKMOL: a package for building initial configurations for molecular dynamics simulations. *Journal of computational chemistry* **30**, 2157-2164 (2009).

5 Hoover, W. G. Canonical dynamics: equilibrium phase-space distributions. *Physical Review A* **31**, 1695 (1985).

6 Parrinello, M. & Rahman, A. Polymorphic transitions in single crystals: A new molecular dynamics method. *Journal of Applied physics* **52**, 7182-7190 (1981).

7 Chiu, S.-W., Pandit, S. A., Scott, H. & Jakobsson, E. An improved united atom force field for simulation of mixed lipid bilayers. *The Journal of Physical Chemistry B* **113**, 2748-2763 (2009).

8 Kučerka, N., Nieh, M.-P. & Katsaras, J. Fluid phase lipid areas and bilayer thicknesses of commonly used phosphatidylcholines as a function of temperature. *Biochimica et Biophysica Acta (BBA)-Biomembranes* **1808**, 2761-2771 (2011).

9 Lewis, B. A. & Engelman, D. M. Lipid bilayer thickness varies linearly with acyl chain length in fluid phosphatidylcholine vesicles. *Journal of molecular biology* **166**, 211-217 (1983).

10 Leekumjorn, S. & Sum, A. K. Molecular characterization of gel and liquid-crystalline structures of fully hydrated POPC and POPE bilayers. *The Journal of Physical Chemistry B* **111**, 6026-6033 (2007).

11 Siu, S. W., Vácha, R., Jungwirth, P. & Böckmann, R. A. Biomolecular simulations of membranes: physical properties from different force fields. *The Journal of chemical physics* **128**, 125103 (2008).

12 Liu, Y. & Nagle, J. F. Diffuse scattering provides material parameters and electron density profiles of biomembranes. *Physical Review E* **69**, 040901 (2004).

13 Kučerka, N. *et al.* Lipid bilayer structure determined by the simultaneous analysis of neutron and X-ray scattering data. *Biophysical journal* **95**, 2356-2367 (2008).

14 Pan, J., Tristram-Nagle, S., Kučerka, N. & Nagle, J. F. Temperature dependence of structure, bending rigidity, and bilayer interactions of dioleoylphosphatidylcholine bilayers. *Biophysical journal* **94**, 117-124 (2008).

15 Hughes, Z. E., Mark, A. E. & Mancera, R. L. Molecular dynamics simulations of the interactions of DMSO with DPPC and DOPC phospholipid membranes. *The journal of physical chemistry. B* **116**, 11911-11923 (2012).

16 Hyvonen, M. T. & Kovanen, P. T. Molecular dynamics simulations of unsaturated lipid bilayers: effects of varying the numbers of double bonds. *European biophysics journal : EBJ* **34**, 294-305 (2005).

17 http://www.scm.com/ADF/.

18 Te Velde, G. *et al.* Chemistry with ADF. *Journal of Computational Chemistry* **22**, 931-967 (2001).

19 Stephens, P., Devlin, F., Chabalowski, C. & Frisch, M. J. Ab initio calculation of vibrational absorption and circular dichroism spectra using density functional force fields. *The Journal of Physical Chemistry* **98**, 11623-11627 (1994).

20 Van Lenthe, E. & Baerends, E. J. Optimized Slater-type basis sets for the elements 1–118. *Journal of computational chemistry* **24**, 1142-1156 (2003).

21 http://manual.gromacs.org/documentation/.

22 Silvius, J. R. Thermotropic phase transitions of pure lipids in model membranes and their modifications by membrane proteins. *Lipid-protein interactions* **2**, 239-281 (1982).
